# *N*-acetylcysteine prevents catheter occlusion and inflammation in catheter associated-urinary tract infections by suppressing urease activity

**DOI:** 10.3389/fcimb.2023.1216798

**Published:** 2023-10-26

**Authors:** Arthika Manoharan, Jessica Farrell, Vina R. Aldilla, Greg Whiteley, Erik Kriel, Trevor Glasbey, Naresh Kumar, Kate H. Moore, Jim Manos, Theerthankar Das

**Affiliations:** ^1^ Infection, Immunity and Inflammation Theme, School of Medical Sciences, Charles Perkins Centre, The University of Sydney, Sydney, NSW, Australia; ^2^ Sydney Institute of Infectious Disease, The University of Sydney, Sydney, NSW, Australia; ^3^ Whiteley Corporation, Tomago, NSW, Australia; ^4^ School of Chemistry, The University of New South Wales, Sydney, NSW, Australia; ^5^ School of Medicine, Western Sydney University, NSW, Australia; ^6^ Department of Urogynaecology, St George Hospital, University of New South Wales, Sydney, NSW, Australia

**Keywords:** *Proteus mirabilis*, UTI, catheter-associated urinary tract infections (CA-UTI), *N*-acetyl cysteine (NAC), urease, biofilms

## Abstract

**Introduction:**

*Proteus mirabilis* is a key pathobiont in catheter-associated urinary tract infections (CA-UTIs), which is well known to form crystalline biofilms that occlude catheters. Urease activity alkylates urine through the release of ammonia, consequentially resulting in higher levels of Mg^2+^ and Ca^2+^ and formation of crystals. In this study, we showed that *N*-acetyl cysteine (NAC), a thiol antioxidant, is a potent urease inhibitor that prevents crystalline biofilm formation.

**Methods:**

To quantify urease activity, Berthelot’s method was done on bacterial extracts treated with NAC. We also used an *in vitro* catheterised glass bladder model to study the effect of NAC treatment on catheter occlusion and biofilm encrustation in *P. mirabilis* infections. Inductively-coupled plasma mass spectrometry (ICP-MS) was performed on catheter samples to decipher elemental profiles.

**Results:**

NAC inhibits urease activity of clinical *P. mirabilis* isolates at concentrations as low as 1 mM, independent of bacterial killing. The study also showed that NAC is bacteriostatic on *P. mirabilis*, and inhibited biofilm formation and catheter occlusion in an *in vitro*. A significant 4-8_log10_ reduction in viable bacteria was observed in catheters infected in this model. Additionally, biofilms in NAC treated catheters displayed a depletion of calcium, magnesium, or phosphates (>10 fold reduction), thus confirming the absence of any urease activity in the presence of NAC. Interestingly, we also showed that not only is NAC anti-inflammatory in bladder epithelial cells (BECs), but that it mutes its inflammatory response to urease and *P. mirabilis* infection by reducing the production of IL-6, IL-8 and IL-1b.

**Discussion:**

Using biochemical, microbiological and immunological techniques, this study displays the functionality of NAC in preventing catheter occlusion by inhibiting urease activity. The study also highlights NAC as a strong anti-inflammatory antibiofilm agent that can target both bacterial and host factors in the treatment of CA-UTIs.

## Introduction

*P. mirabilis* is a Gram-negative pathogen frequently isolated from patients with catheter-associated urinary tract infections (CA-UTIs) and complicated UTIs (Allison et al., 1994; Chen et al., 2012). CA-UTIs are the most common healthcare-associated infection (HAI), affecting approximately 150-200 million patients worldwide annually (Zowawi et al., 2015). *P. mirabilis* accounts for almost 10% of all hospital-associated UTIs worldwide (Schaffer and Pearson, 2015) and 45% of all CA-UTIs (Stickler, 2008; Nzakizwanayo et al., 2016). The indicative global mortality rate for geriatric CA-UTIs patients infected with *P. mirabilis* is 45% (Schaffer and Pearson, 2015).*P. mirabilis* is one of the most challenging uropathogens to eradicate, given its ability to self-elongate and produce substances that express proteins which facilitate adhesion to and motility on catheters. Infection with *P. mirabilis* is often severe and can result in permanent renal damage, sepsis and death in some cases (Armbruster et al., 2018).

Bacteria are often introduced into the bladder from the perianal area near the urethral opening (which is heavily colonised by gut microbiota) or by contaminated catheters that are not aseptically handled (Pelling et al., 2019). *P. mirabilis* is often found in commensal gut flora (Armbruster et al., 2018), which was demonstrated by Mathur et al. (2005), where in many patients, the CA-UTI causing isolates of *P. mirabilis* matched those in their faecal samples (Mathur et al., 2005). *P. mirabilis* has the greatest ability of all Gram-negative uropathogens to attach to catheters (Roberts et al., 1990), primarily attributed to its numerous fimbriae and the many adhesins it possesses (Armbruster et al., 2018). In the catheter lumen, *P. mirabilis* forms a distinct crystalline biofilm as a result of its urease activity in the presence of the constant urine flow. Urine provides numerous constituents, including host proteins, that facilitate attachment and biofilm formation (Pelling et al., 2019).

Urease is a nickel-dependant metalloenzyme produced by *P. mirabilis* and other bacterial species and its expression is induced at body temperature (37°C) and in high urea concentrations (Poore and Mobley, 2003). The high concentrations of urea present in urine (approximately 400 mM) implies that *P. mirabilis* urease is constitutively expressed (Coker et al., 2000). Urease hydrolyses urea to produce ammonia and carbon dioxide, facilitating the alkalinisation of urine (through increasing ammonia concentrations) and consequential precipitation of divalent ions (Mg^2+^, Ca^2+^). The enzyme is found in the cytoplasm as well as on the outer cell membrane (McLeanl et al., 1986) and is a driving factor in catheter colonisation. This is because it allows for bacterial utilisation of nitrogen as a nutrient source, thus promoting enhanced bacterial health and survival. This leads to the precipitation of struvite (magnesium ammonium phosphate) and apatite (calcium phosphate) crystals on the catheter lumen (Coker et al., 2000), together with increased bacterial adhesion and crystalline biofilm formation (Stickler et al., 2006).

Crystalline biofilms can rapidly obstruct the catheter and block urine flow, resulting in backflow and accumulation of infected urine in the bladder and further up the urinary tract. This disrupts normal kidney function and results in severe complications such as pyelonephritis and septicaemia (Stickler, 2014). Furthermore, the crystals can also cause trauma to the bladder mucosa and the urethra while also providing a foundation for bladder stone formation and reinfection (Stickler, 2008). 50% of all patients undergoing long-term catheterisation will experience catheter occlusion (Mathur et al., 2006; Nzakizwanayo et al., 2016).

The crystalline nature of the biofilm further exacerbates antibiotic resistance of communities within these biofilms as studies have shown greater protection is afforded towards bacteria against antimicrobials by this mineralisation (Li et al., 2016). The study also demonstrated that crystallisation accounts for 78% of the biofilm matrix at the 24 h timepoint when *P. mirabilis* biofilm is formed in artificial urine (Li et al., 2016).

Inhibiting urease activity significantly decreases *P. mirabilis* virulence (Abdel-Baky et al., 2017), as urease-negative *P. mirabilis* mutants cannot maintain high survival and colonisation in the bladder unlike urease-expressing strains. More interestingly, urease positive *P. mirabilis* outcompetes urease-negative or less urease-positive pathogens in coinfections, even when introduced 72 h later (Armbruster et al., 2014; Armbruster et al., 2017). Therefore, targeting urease expression as a therapeutic modality could reduce crystallisation from urease activity and consequently decrease the robustness of biofilms formed, thus allowing for greater biofilm eradication.

Current treatment options for *P. mirabilis* CA-UTIs are limited to antibiotic therapy, such as ciprofloxacin (Wasfi et al., 2020), and catheter replacement in cases of catheter blockage. However, catheter replacements are painful for the patient, and bacteria in bladder biofilms (often surviving in very high titres) can rapidly colonise these newly inserted catheters (Milo et al., 2021).

Another viable option is urease inhibition. Currently acetohydroxamic acid (AHA) is the only inhibitor approved by the US FDA and EU regulators. AHA competes for urease binding sites and can be used in patients with *P. mirabilis* UTIs or *Helicobacter pylori* infections (Kappaun et al., 2018; Milo et al., 2021). However, AHA causes numerous severe side effects including teratogenesis and haemolytic anaemia, which have reduced its clinical use (Williams et al., 1984; Kappaun et al., 2018), therefore creating an urgent need for an alternative urease inhibitor.

*N*-acetyl cysteine (NAC) is a thiol antioxidant that is the precursor of glutathione synthesis in mammalian cells. The anti-biofilm effects of NAC on various biofilm-forming pathogens from the lungs, skin, urinary tract and elsewhere are well described (Perez-Giraldo et al., 1997; Marchese et al., 2003; Zhao and Liu, 2010; Blasi et al., 2016; Manoharan et al., 2020; Aiyer et al., 2021; Manoharan et al., 2021). The antibiofilm action of NAC is hypothesised to arise from its thiol moiety, with research to confirm this still under way. NAC has demonstrated efficacy in preventing biofilm formation on both biotic and abiotic surfaces (Costa et al., 2017). We have also recently shown NAC displays no cytotoxicity against bladder cells *in vitro* (Manoharan et al., 2021), thus making NAC a safe candidate for UTI therapy.

In this study we first investigated the anti-urease effect of NAC on several clinical *P. mirabilis* isolates at both low and higher MIC concentrations. In parallel, we sought to identify the effect of NAC on biofilm formation and biofilm bacteria in catheters *in vitro*. Importantly, we also determined the effect of NAC treatment on host inflammatory responses to urease and *P. mirabilis* infection. Finally, we aimed to elucidate if urease inhibition by NAC can prevent catheter occlusion *in vitro*. Together, this study describes the powerful potential of NAC treatment in addressing both host and pathogen factors that contribute to biofilm formation and pathogenesis during CA-UTIs.

## Materials and methods

### Reagents and media used in this study

NAC, ciprofloxacin, trypsin-EDTA, gentamicin, Triton X-100, resazurin, glycerol, penicillin/streptomycin solution and kanamycin were purchased from Sigma-Aldrich, Sydney, Australia. Tryptic soy broth (TSB) and Tryptone soy agar (TSA) were obtained from Oxoid (Thermo-Fisher, Scoresby, Australia). 1× phosphate-buffered saline (PBS) was purchased from POCD, Australia. RPMI 1640 media containing L-glutamine, sodium bicarbonate was obtained from Lonza (Sydney Australia). Foetal Bovine Serum (FBS) was purchased from Thermo-Fisher. Alexa Fluor^®^ 647 conjugated Wheat Germ Agglutinin (WGA) and 4′,6-diamidino-2-phenylindole (DAPI) nucleic acid stain were obtained from Invitrogen, (Thermo-Fisher, Scoresby, Australia).

### Composition of artificial urine medium

The artificial urine medium was prepared according to the method of Brooks and Keevil (1996) (Brooks and Keevil, 1997), with the composition of individual components listed in **Table 1**.

**Table 1:** Components of artificial urine media used in this study.

Component	Concentration	Source
**Urea**	25 g/L	Sigma-Aldrich (Sydney, Australia)
**Calcium hydroxide**	2.49 g/L	Ajax chemicals (Sydney, Australia)
**Sodium sulphate**	3.2 g/L	Sigma-Aldrich (Sydney, Australia)
**Sodium chloride**	5.2 g/L	Sigma-Aldrich (Sydney, Australia)
**Magnesium sulphate**	0.49 g/L	Sigma-Aldrich (Sydney, Australia)
**Iron sulphate heptahydrate**	0.0006 g/L	Sigma-Aldrich (Sydney, Australia)
**Ammonium chloride**	1.3 g/L	Ajax chemicals (Sydney, Australia)
**Yeast extract**	0.005 g/L	Sigma-Aldrich (Sydney, Australia)
**Lactic acid**	0.05 g/L	Sigma-Aldrich (Sydney, Australia)
**Citric acid**	0.4 g/L	Ajax chemicals (Sydney, Australia)
**Potassium dihydrogen phosphate**	0.95 g/L	Ajax chemicals (Sydney, Australia)
**Dipotassium hydrogen phosphate**	1.2 g/L	Ajax chemicals (Sydney, Australia)
**Creatinine**	0.25 g/L	Sigma-Aldrich (Sydney, Australia)
**Sodium bicarbonate**	2.1 g/L	Sigma-Aldrich (Sydney, Australia)
**HCl**	1 M	Ajax chemicals (Sydney, Australia)
**NAC**	30 mM	Sigma-Aldrich (Sydney, Australia)

### Composition of Stuart’s broth

The baseline composition is described in **Table 2**. The concentration of urea in the broth was varied between 3 to 25 mM and the concentration of NAC in the broth was varied between 1mM to 50 mM, depending on requirements.

**Table 2:** Composition of Stuart’s broth.

Component	Concentration	Source
**Urea**	3-25mM	Sigma-Aldrich (Sydney, Australia)
**Dipotassium hydrogen phosphate**	4.75 g/L	Ajax chemicals (Sydney, Australia)
**Potassium dihydrogen phosphate**	4.55 g/L	Ajax chemicals (Sydney, Australia)
**Yeast extract**	0.05 g/L	Sigma-Aldrich (Sydney, Australia)
**Phenol-red**	0.005 g/L	Thermo-Fisher, Australia

### Cell culture

The human bladder epithelial cell line (BECs) 5637 (ATCC 5637) (https://www.atcc.org/products/htb-9) stocks were stored in liquid nitrogen in Foetal Bovine Serum FBS containing 20% (v/v) dimethyl sulfoxide (DMSO). For all downstream uses, cell stocks were resuscitated and cultured in RPMI 1640 (media supplemented with 10% (v/v) FBS and 1% (w/v) penicillin/streptomycin at 37°C in a humidified atmosphere containing 5% (v/v) CO_2._ For artificial urine media assays, RPMI media supplemented with 10% (v/v) FBS was used.

### Determining the minimum inhibitory concentrations of NAC/ciprofloxacin on planktonic *P. mirabilis*

As described before (Manoharan et al., 2020), MICs for *P. mirabilis* were determined using the broth dilution method. In brief, overnight grown cultures in TSB (at 37°C and 150 rpm) were centrifuged at 5000 xg for 5 min, following which supernatant was decanted and bacteria were resuspended in fresh TSB. Cultures were then inoculated at OD_600_ = 0.1 ± 0.02 into 96-well, flat-bottomed plates (Corning Corp., USA) in the presence of NAC (0–30 mM) at *t *=* *0. Plates were then incubated for 48 h at 37°C and 100 rpm and OD_600nm_ was recorded 48 h post incubation using a plate reader (Tecan Infinite M1000 pro). Percentage bacterial growth was calculated, and the MIC was determined to be the concentration at which most strains showed >60% reduction in bacterial growth.

### Identifying the bacteriostatic/ bactericidal effect of NAC on *P. mirabilis*

Following previously published methods (Manoharan et al., 2020), we sought to establish whether the antibacterial effect of NAC observed on *P. mirabilis* was bactericidal or bacteriostatic. Briefly, the bactericidal effect of NAC was determined by inoculating cultures of OD_600_ = 0.1 ± 0.02 into 96-well, flat-bottomed plates in TSB in the presence of NAC, measuring OD_600_ at various timepoints for 48 h. Plates were then incubated for 48 h at 37°C and 100 rpm. Following the 48 h incubation, plates were centrifuged at 4500 x**g** for 15 mins. The supernatant was then discarded and 1× PBS was used as a wash. Plates were centrifuged again at 4500 x**g** for 15 mins and any remaining bacteria in the wells were resuspended in fresh TSB. Growth of bacteria was then monitored by measuring OD_600_ for 24 h. Percentage bacterial revival was calculated to determine bacteriostatic or bactericidal effect.

### Determining the minimum biofilm inhibitory concentration (MBIC) of NAC/antibiotic combinations on bacterial strains

MBICs were done as described previously (Manoharan et al., 2021). Briefly, overnight bacterial cultures at a density of OD_600 nm_ = 0.1 ± 0.02 were added to 96-well tissue culture plates in PBS and allowed to adhere for 1 h at 37°C, 100 rpm in an orbital shaker. Wells were washed with 1× PBS to remove any unadhered bacteria. TSB containing varying concentrations of NAC was added and plates incubated for an additional 48 h. Biofilm formation was measured by measuring bacterial density at OD_600nm_ using a plate reader (Tecan infinite M1000 pro, Melbourne, Australia).

### Quantifying the effect of NAC on initial attachment of bacteria to polymer substratum

The effect of NAC on bacterial adhesion was measured using an initial adhesion assay as previously described (Manoharan et al., 2020; Manoharan et al., 2021). Briefly, six-well plates (Corning Corp., Corning, NY, USA) were seeded with bacteria at a density of OD_600 nm_ = 0.1 ± 0.02 and incubated for 2 h at 37°C and 100 rpm. Plates were then washed twice with 1× PBS and imaged using phase contrast microscopy at 200× magnification (Zeiss AxioScope.A1 FL, LED, Jena, Germany). Bacterial counts were performed on images using Fiji, Image J (https://imagej.nih.gov/ij/plugins/cell-counter.html).

### Quantification of bacterial load in treated biofilms

Preformed biofilms were grown by inoculating 96-well, flat-bottomed plates with overnight bacterial cultures at OD_600_ = 0.1 ± 0.02. Plates were then incubated for 48 h in TSB at 37°C and 100 rpm to allow for biofilm growth, washed once with 1× PBS and treated as described in **Table 3** followed by incubation for 24 h. Controls were incubated in the presence of 1x PBS alone for the duration of incubation. The treatment was subsequently removed by washing once with 1× PBS and any biofilms formed were scraped from the surface and homogenised in 200 µL 1× PBS. Serial dilutions of scrapings were plated onto TSA plates and incubated at 37°C overnight. Colonies were enumerated and expressed as CFU/mL.

**Table 3:** Combination of treatments tested in this study.

Combinations of treatments tested	NAC (mM)	Ciprofloxacin (mg/L)
	20	–
	30	–
	40	–
	50	–
	30	6
	–	6

### Tagging clinical *P. mirabilis* isolates with green fluorescent protein (GFP) for bacterial visualisation

To visualise bacterial invasion of BECs a clinical isolate of *P. mirabilis* was tagged with green fluorescent protein (GFP) using a published protocol (Manoharan et al., 2021) with free use GFP gene (fuGFP) on the pU252 plasmid. pUS252 was a gift from Nicholas Coleman (Addgene plasmid # 127674 ; http://n2t.net/addgene:127674; RRID:Addgene_127674). The fuGFP contains a kanamycin marker in a gene cassette as a selective criterion and as such, the MIC of kanamycin for the clinical strains had to be established. Isolate *P. mirabilis* 44 was determined to be kanamycin sensitive, and thus chosen to be tagged with fuGFPb. Overnight cultures were made electrocompetent by centrifugation (5000 *g*, 4°C for 15 min). The supernatant was discarded, and the pellet was resuspended in 50 mL of cold sterile 10% v/v glycerol and centrifuged (5821 *g*, 4°C for 15 min). The pellet was then resuspended in 5 mL of 10% (v/v) glycerol again and stored at −80°C prior to transformation. Plasmid DNA containing fuGFPb and kanamycin resistance was added to 50 μL of bacteria, the mixture was transferred to an electroporation cuvette and subjected to 2500 V, 25 µF, and 200 Ω of current for electroporation (Gene Pulser Xcell Microbial System, BIO-RAD, Hercules, CA, USA) followed by incubation for 1 h at 37°C at 150 rpm. Following this, the transformed bacteria were plated onto Luria–Bertani (LB) agar containing 250 μg/mL kanamycin to select for successfully transformed bacterial colonies.

### Determination of NAC cytotoxicity using ISO standard methods

Cytotoxicity testing was performed following the ISO method (British Standard ISO 10993-5:2009)—direct contact assay. In brief, six-well plates were seeded with cells at a density of 10^6^ cells/mL and allowed to grow to confluence for 48 h. At approximately 90% confluence, cells were washed with PBS and an antibiotic filtered disc (6mm, Whatman GE Healthcare, Sydney, Australia), impregnated with NAC was placed into the centre of the well. RPMI with 10% FBS only was then added, and plates incubated for 24 h. Plates were subsequently washed using 1 x PBS and imaged using phase contrast microscopy. For determination of respiring cell levels, plates were incubated in the presence of 0.05% w/v resazurin in PBS for 24 h, and fluorescence intensity was measured using a plate reader (Tecan Infinite M1000 pro; Ex/Em at 544/590 nm).

### Visualisation of biofilm architecture following combination treatment

Biofilms were grown by inoculating 4-chambered glass slides with 1 mL of overnight bacterial culture at OD_600_ = 0.1 ± 0.02 in TSB and were incubated for 48 h at 37°C under static conditions. Slides were gently washed once with 1× PBS to remove unattached bacteria and treated with a combination of NAC and ciprofloxacin for 24 h. Biofilms were washed once with 1× PBS and stained with a live/dead stain (Bacterial Viability Kit, Molecular Probes, Thermo-Fisher Scientific, USA), followed by incubation in the dark for 45 min before imaging. Biofilms were visualised by Confocal Laser Scanning Microscopy (CSLM) (Olympus FV1200, Australia) with Ex/Em 473/559 nm and Ex/Em 500/637 nm for Syto 9 (green for live cells) and PI (red for damaged and dead cells) staining, respectively. All biofilms were imaged at 400× magnification with 3D proportions. Images were obtained and analysed using Fiji ImageJ software (USA).

### Measurement of bacterial migration through catheter tubing using a catheter bridge model

A catheter bridge model was used as previously published (Jones et al., 2004; Kazmierska et al., 2010) to assess NAC's effectiveness in preventing bacterial migration through catheters. Size 14-Foley catheters were cut into 1cm long sections impregnated with 0-, 30- or 50-mM NAC in the presence of 100% (v/v) chloroform (Sigma Aldrich, Sydney, Australia).

One cm-wide channel was cut across TSA plates (Thermo-Fisher), and 1 µL of overnight-grown culture was inoculated onto one agar shore. Catheter segments were then placed into the channel after being dried, and plates were incubated for 24 h at 37°C. Controls with no bridge and one side of inoculated agar were used to establish that bacteria could only migrate to the other side of the plate through the lumen of the catheter bridge. Following incubation, catheter bridges were scraped, homogenized in 1xPBS, sonicated at 1500 Hz, serially diluted and plated on TSA to enumerate the bacteria in the “bridge”, as CFU/mL. Migration through the catheter bridge was established by growth on the other uninoculated side of the bridge. Any bacterial growth on the uninoculated side was confirmed in two ways. First, 1 cm x 1 cm squares of agar were excised, inoculated into TSB and incubated in an orbital shaker at 150 rpm and 37°C overnight. Absorbance at OD_600nm_ was then measured using the spectrophotometer to quantify any presence of bacterial growth. Additionally, swab plates of the uninoculated side were done, with swabs placed in TSB and incubated overnight at 37°C, following which the presence or absence of growth was recorded. Images of agar plates were taken using a mobile phone for visual observation.

### Quantification of bacterial urease activity

To quantify remnant urease activity post-treatment, Berthelot’s method (Weatherburn, 1967) was adapted and slightly modified. First, overnight-grown bacterial cultures were centrifuged at 3715*g* for 10 min. The pellet was washed by two rounds of resuspension in 15 mM K_2_HPO_4_ and centrifugation at 4851*g* for 10 min. The pellet was then resuspended in 15 mM K_2_HPO_4_ to an OD_600nm_=0.7± 0.05. Bacterial suspensions were exposed to 0.5 mM, 1 mM, 5 mM, 10 mM, 30 mM or 50 mM NAC in a total volume of 5 mL, and incubated with orbital shaking at 150 rpm, 37°C for 2 h. After incubation, bacteria were centrifuged as above and washed three times to remove any treatment. The pellet was resuspended in 3 mL of K_2_HPO_4_ in a 15 mL falcon tube. The bacteria were then sonicated for 90 seconds at 50% amplitude using an ultrasonic water bath sonicator.

The sonicated bacterial lysates were then tested for urease activity using the Berthelot’s method as follows: 300 μL of sonicated bacterial lysate was added to 200 μL of 25 mM urea and incubated in a water bath at 37°C for 30 min. 500 μL each of three solutions: one containing 10g/L phenol, 50g/L sodium nitroprusside and two containing 5% (v/v) sodium hypochlorite and 5g/L sodium hydroxide, were added and the lysate incubated for an additional 15-30 min until a colour change was observed. The absorbance of samples was measured at 625 nm and used as a measure of urease activity.

To eliminate the effect of bacterial killing (if any) on urease inhibition activity, overnight cultures of bacteria were also treated as described above. Following treatment, bacteria were washed twice by centrifuging cultures at 2851 *g* for 10 min. Bacteria were resuspended in PBS and plated onto TSA plates for bacterial enumeration.

### Kinetics of Jack Bean Urease and its inhibition by NAC

Kinetic assays on urease were performed in Stuart’s broth (Milo et al., 2021) containing phenol red indicator. 100 μL of Stuart’s broth with urea ± NAC was added to 100 μL of 200 μg/mL Jack Bean Urease (JBU). Absorbance was measured for 10 cycles of 30 seconds at OD_560nm_ using spectrophotometry. To determine the Michaelis-Menten constant, the experiment was performed with three different concentrations of urea (3 mM, 5 mM and 25 mM) and five different concentrations of NAC (1 mM, 5 mM, 10 mM, 30 mM, 50 mM). The initial reaction rate was then determined, and kinetic values were predicted using GraphPad Prism 8.0.0 (USA).

### The effect of NAC on the conformation and structure of JBU

To elucidate the effect of NAC on the structure of urease and its conformation, circular dichroism spectroscopy was performed. 500 μg/mL JBU was incubated with different concentrations of NAC below 0.5 mM for 1 h at 100 rpm in an orbital shaker. Samples were then run on a Chirascan Plus CD spectrometer (Applied Photophysics, UK) in a cuvette with a 1 mm path length at ambient temperature. Data was collected between 180 nm – 300 nm wavelength, at a step of 1 nm with 0.5 seconds per point. Urease and NAC were both dissolved in deionised water. The final spectra were baseline, background corrected and presented as the mean of residue ellipticity.

### Effect of NAC on catheter encrustation *in vitro*

**Figure 1:**
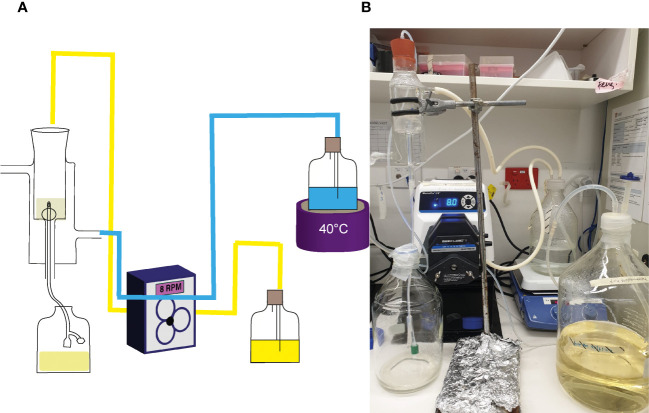
Laboratory set up of *in vitro* glass bladder model. **(A)** Schematic of the glass bladder model arrangement. Arrows depict the flow of artificial urine media through the bladder. The blue lines represent the flow of water through silicon tubes into the glass jacket around the bladder compartment, while the yellow represents flow of AUM through silicon tubes into the bladder. **(B)** Photo showing the actual arrangement in use.

The efficacy of NAC in preventing catheter encrustation and blockage was investigated using an *in vitro* glass bladder model (Stickler et al., 1999; Stickler and Jones, 2008; University of Sydney Wc, 2022). The double-jacketed water-filled glass bladder was maintained at 37°C, into which a size 14 Foley catheter was inserted (**Figure 1**). The catheterised bladder was then inoculated with 10^8^ CFU/mL of *P. mirabilis* to emulate a late-stage CA-UTI and incubated at 37^o^C for 2 h to allow bacterial adhesion. Artificial urine media with/without NAC (30mM) or AHA (10mM) wan pumped through the bladder at a rate of 29 mL/h to simulate sheer force. Time to blockage was then measured, at which point the artificial urine media supply was ceased. In the event of no visible blockage, experiments were terminated at the 120 h time point. Urine within the bladder was also sampled at 24 h intervals until the experimental endpoint. Catheters were harvested, cut into 1 cm sections, and washed twice in 1x PBS. The sections were then evenly scraped 30 times on each side using a wooden toothpick and homogenised into 3 mL of 1 x PBS in a falcon tube. The catheter section and toothpick were then sonicated for 15 min at 50% amplitude, following which the solutions were plated onto TSA plates for bacterial enumeration. Catheters were sampled at 3, 5 and 10 cm below the catheter eye (opening), alongside the eye itself.

### The effect of NAC on the elemental profile of biofilms formed in catheters *in vitro*

To assess if urease inhibition and NAC treatment influenced the elemental profiles of crystal deposits in catheters, the eye of catheters harvested were subjected to Inductively Coupled Plasma Mass Spectrometry (ICP-MS) (Perkins-Elmer, Australia). Encrusted biofilms in the catheter eye were dissolved in 1 mL 10% (v/v) nitric acid and sonicated for 10 mins to aid crystal removal and biofilm disruption. Samples were then held for 24 h at room temperature to allow crystals to dissolve. The samples were then centrifuged for 30 seconds on a spin top centrifuge and further diluted to 1% (v/v) nitric acid before being run on the ICP-MS machine. The elemental panel looked for the presence of 12 different elements, out of which seven were highlighted for the purpose of this study.

### Quantification of inflammatory IL-6, IL-8, IL-1b and TNF-a responses to urease and *P. mirabilis* infection post NAC treatment

The inflammatory responses of BECs to the presence of urease, with and without added NAC, were quantified using commercial ELISA kits. 5637 BECs were seeded in 12 well plates at a density of 1x10^5^ cells and allowed to grow to confluence. Cells were then exposed to NAC/urease combinations for 24 h. Cellular supernatants were then collected, centrifuged at 10000*g* for 10 min and stored at -80°C until ELISAs were performed.

Inflammatory responses to bacterial infection in the presence and absence of NAC were also quantified. 5637 BECs were seeded as described above, and confluent cells were infected with *P. mirabilis* strain 67 at an OD_600nm_= 0.1 ± 0.02 (~10^6^ CFU/mL), and the plate was centrifuged at 120*g* for 2 min to initiate bacterial contact. Cells were then incubated for 2 h, following which cellular supernatants were collected, centrifuged at 10000*g* for 10 min and stored at *-*80°C.

Commercial ELISA kits were obtained from Abcam (Cambridgeshire, United Kingdom) (**Supplementary Table 2**) and ELISA tests were performed according to the manufacturer’s instructions.

### Quantifying bacterial invasion of bladder epithelial cells in the presence of NAC

To quantify the effect of NAC on bacterial invasion, bacterial invasion assays in the presence of NAC were performed as previously described (Manoharan et al., 2021). BECs were seeded into 12-well tissue culture plates at a density of 5 × 10^5^cells/mL and allowed to grow to 95% confluence. Cells were then pre-treated with NAC 2 h before infection. Overnight grown *P. mirabilis* cultures were centrifuged and the bacterial pellet was resuspended in RPMI supplemented with 10% (v/v) FBS to an OD_600_ = 0.1 ± 0.02. BECs were then infected with bacteria at a multiplicity of infection (MOI) of 10 for 2 h in the presence of NAC.

The BECs were washed to remove any non-adherent bacteria and treated with 1 mg/mL gentamicin for 1 h to remove any extracellular bacteria. Cells were then lysed with 0.5% (v/v) Triton-X 100 and serial dilutions plated onto TSA plates to enumerate intracellular bacterial loads, expressed as CFU/mL. *P. mirabilis* 67 was tested in invasion assays as a model strain used throughout this study, while *P. mirabilis* 44 was also tested, given that it was the only kanamycin-sensitive strain that could be used to visualise bacterial invasion to complement these invasion assays.

### Visualisation of inhibition of bacterial invasion of bladder epithelial cells

To provide visual support to the invasion assay above, our clinical model strain for *P. mirabilis* was tagged with fuGFPb (as described) to track its intracellular bacterial colony formation using fluorescence microscopy. This was carried out as previously described (Manoharan et al., 2021). Cells were seeded in a 4-well chamber slide at a density of 10,000 cells/mL and incubated for 48 h to grow to confluence. Cells were infected with fuGFPb-tagged *P. mirabilis* strain 44 following the invasion assay protocol described above. The cells were stained with Alexa fluor-647 conjugated WGA (Invitrogen, Thermo-Fisher) for 10 min to stain the cytoskeleton (WGA binds to glycoproteins of the cell membrane), and then counterstained with DAPI (Invitrogen, Thermo-Fisher) for 1 minute. Slides were fixed with 10% (w/v) paraformaldehyde and imaged using a Confocal Laser Scanning Microscope (CLSM, Olympus FV1200, Melbourne, Australia).

### Statistical analysis

All statistical analysis was performed using GraphPad Prism 8.0.0 (GraphPad, San Diego, CA, USA). Dunnett’s T3 multiple comparisons test was performed for experiments in which multiple comparisons were done and compared to a control group (n<50 in all groups). Tukey’s or Sidak’s multiple comparisons test was performed in experiments in which all tested conditions were compared for significance.

## Results

### Section I: Inhibition of urease activity using lower concentrations of NAC

This section detailed the efficacy of low concentrations of NAC in inhibiting urease activity.

### NAC inhibits *P. mirabilis* urease activity

**Figure 2:**
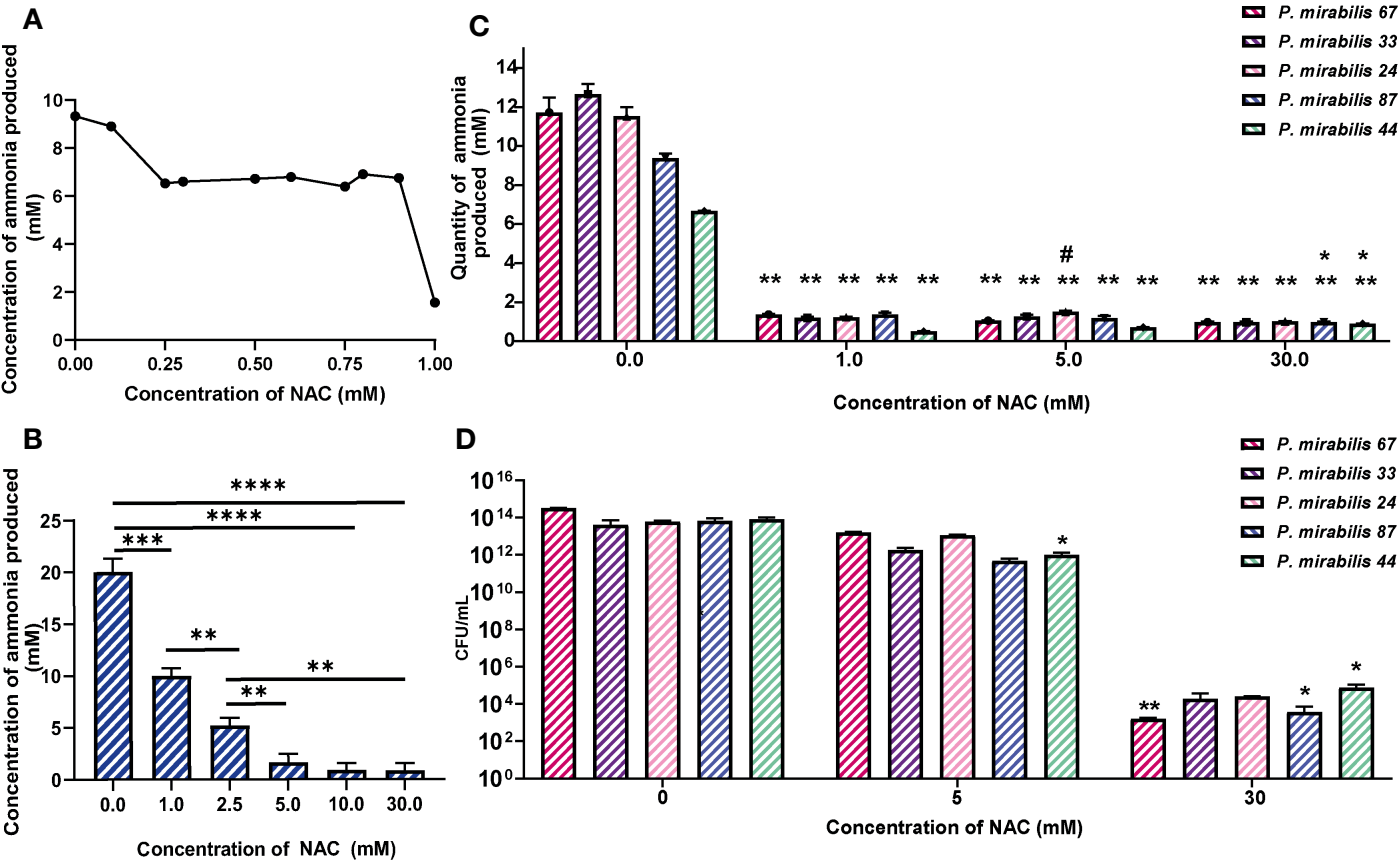
NAC inhibits the activity of urease from clinical *P. mirabilis* isolates and JBU. **(A)** The minimum urease inhibitory concentration was identified using *P. mirabilis* 67 as a representative strain. **(B)** The urease inhibitory concentrations tested on Jack bean urease (JBU) as a control urease. Tukey’s multiple comparisons test was used for statistical analysis, **p<0.01 compared to the respective untreated control, *p<0.05 compared to the respective untreated control. ****p<0.0001, ***p<0.001. **(C)** The quantity of ammonia produced was used as a measure of urease activity after 2 h exposure to NAC of *P. mirabilis* clinical isolates. Tukey’s multiple comparisons test used for statistical analysis, **p<0.01 compared to respective untreated controls, *p<0.05 compared to 0.5 mM #p<0.05 compared to 5 mM. **(D)** Bacterial counts enumerated as CFU/mL corresponding to data in **(C)** Tukey’s multiple comparisons test used for statistical analysis, *p<0.05, **p<0.01. Data represents the mean of n=5 replicates.

NAC showed maximum urease inhibitory capacity at concentrations as low as 1 mM NAC (over 30 times below the MIC), where ammonia produced by remnant urease activity was significantly reduced by 3-6-fold across all five tested isolates (**Figures 2A, C**). Urease activity at 10 and 30 mM was not much lower than that at 5 mM. To eliminate the possibility of bacterial killing masking any specific urease inhibitory activity by NAC, viable bacteria present were enumerated in the sample before performing Berthelot’s treatment. This showed that at 1/5 MIC (5 mM) of NAC, there was no reduction in *P. mirabilis* bacterial growth (**Figure 2D**), despite the significant decrease in urease activity observed at the same concentration (**Figure 2A**). By comparison, at 30 mM NAC, a significant 10log_10_ reduction in CFU/mL was observed (p<0.01 for *P. mirabilis* 67, p<0.01 for *P. mirabilis* 87). In the case of *P. mirabilis* 44, a significant but lower 8log_10_ reduction in CFU/mL was recorded (p<0.01). **Figure 2B** displays the effect of NAC on JBU, used as a model urease in this study. A concentration-dependent effect was recorded on the quantity of ammonia produced in the presence of NAC. At 5 mM, an almost 5- fold reduction in activity was observed.

### Influence of NAC on enzymatic kinetics of JBU

**Figure 3:**
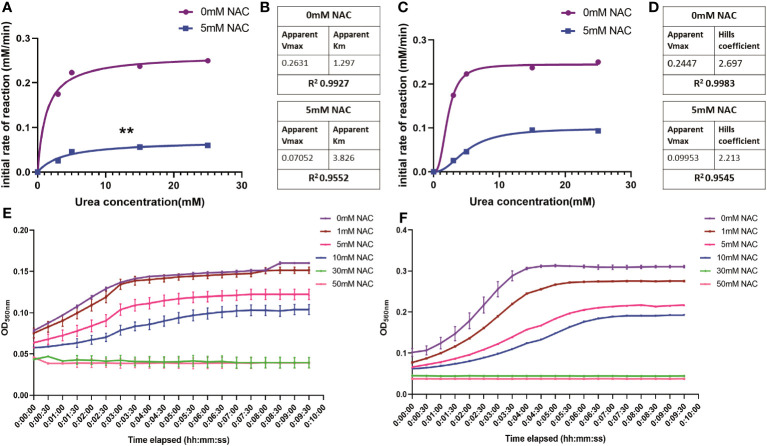
The influence of NAC on the rate of urea degradation by Jack Bean urease. **(A, B)** Hyperbolic curve fitting (Least squares fit) was used to determine Michaelis-Menten parameters and change in V_max_ of JBU and its substrate, urea, in the presence of NAC. **(B)** A summary of these parameters. **(C, D)** Sigmoidal curve fitting to determine the Hills coefficient of urea binding to JBU in the presence of NAC. **(E)** The urea breakdown rate by JBU over 10 minutes, recorded at 15mM urea in the presence of varying concentrations of NAC. **(F)** The urea breakdown rate by JBU over a 10-minute period, recorded at 25mM urea in the presence of varying concentrations of NAC. Data represents the mean of n=3 replicates. The error bars show standard deviation in absorbance values. **p<0.01.

To study the rate of ureolytic activity in the presence of NAC, enzyme kinetics studies were performed using purified JBU. JBU was used given the high level of conserved similarity between *P. mirabilis* urease and JBU and the common conserved active site between the two (Milo et al., 2021). In the presence of NAC, the apparent V_max_ decreased significantly (p<0.01) to 0.07 mM from 0.26 mM, suggesting a solid reduction in the rate of reaction of JBU in the presence of NAC (**Figures 3A, B**). The sigmoidal and hyperbolic curves used to obtain Michaelis-Menten parameters showed a strong fit and correlation (with a control R^2^ value of 0.99).

Furthermore, the Michaelis-Menten constant was determined to be 3.826 mM in the presence of NAC, compared to 1.29 when untreated (**Figure 3B**). This is still within the range of parameters associated with JBU, which is between 2.71-8.81 mM (Chang et al., 2021). The Hills coefficient (which predicts cooperativity in a binding process) was determined to be 2.697 in untreated samples and 2.213 in NAC treated urease (**Figures 3C, D**). This suggests the cooperative binding of substrates in more than one site on the enzyme. Additionally, the reduction in the rate of reaction in the presence of NAC is shown at two different concentrations of urea (**Figures 3E, F**, respectively), with the change in rate of reaction more apparent at 25 mM urea (**Figure 3F**). No urease activity is noted for either concentration of urea at 30- or 50-mM NAC.

### Change in urease conformation in the presence of NAC

**Figure 4:**
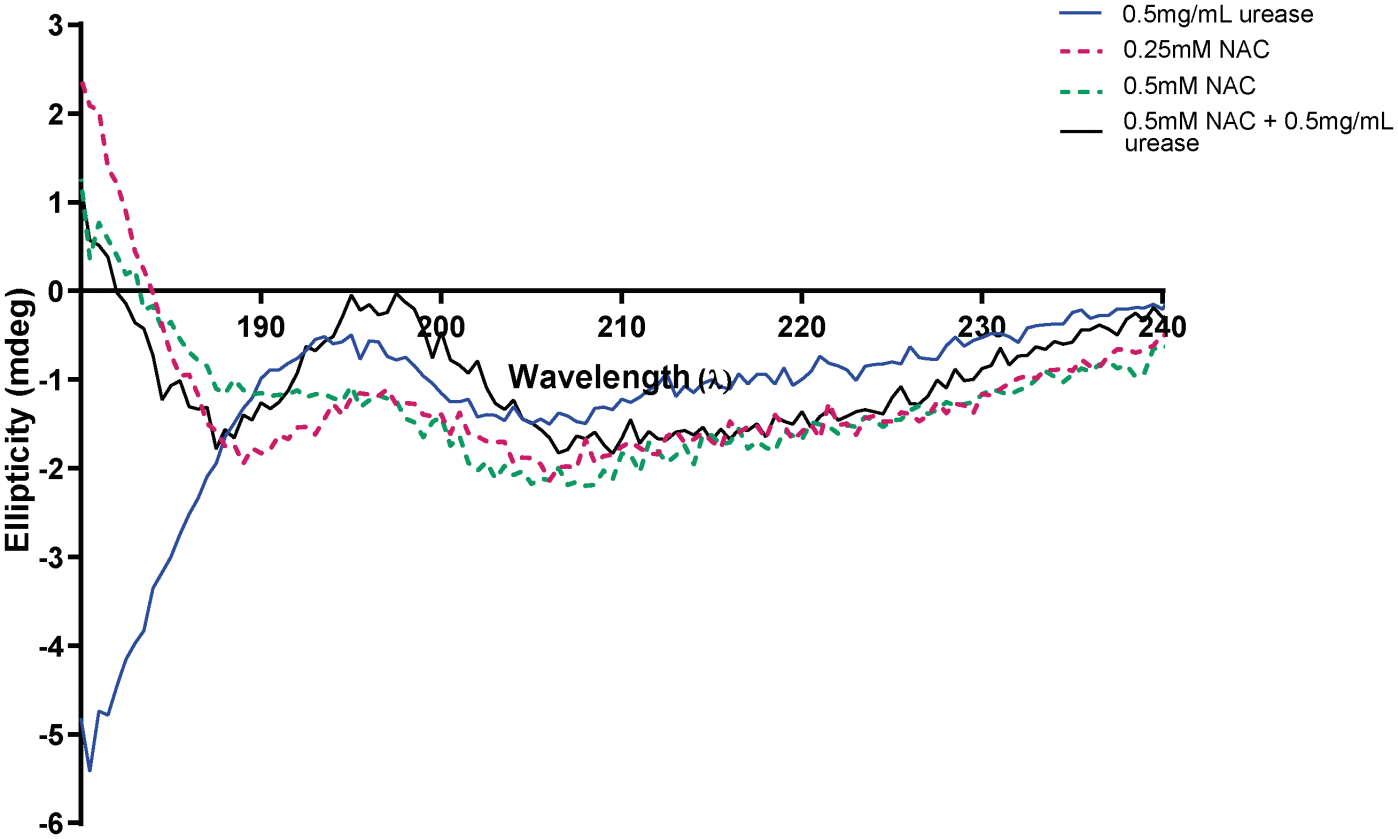
NAC affected the structure and ellipticity of urease. The appearance of the far ultraviolet region of the CD spectrum of urease when treated with NAC (in black). The far-UV region of the CD spectra of NAC and urease alone are also displayed (in red, green, and blue).

At a low concentration of 0.5 mg/mL urease, the enzyme displayed low ellipticity, with a random coil present in the negative ellipticity near 190 nm, a maximum at 195 nm and a minimum at around 205 nm. This is suggestive of a disordered/unfolded protein with an alpha helix structure (around 205-210 nm) (blue) (**Figure 4A**) (Greenfield, 2006). The maxima of NAC at 195 nm displayed a hyperchromic shift (towards zero) in the presence of urease. In addition, a concentration-dependent effect was observed on the random coil in NAC treated urease (**Supplementary Figure 1**). Additionally, other signatures for urease (the tail below 190 nm and peak at 190 nm) disappeared, with a clear peak present at around 199 nm (potentially stemming from the L-cysteine group) (Picot et al., 2019) and the appearance of a minimum at around 188 nm.

All experiments from this point forward use 30 mM NAC. This is because the second aim of the study focused on establishing the anti-urease and antibiofilm effect of NAC on *in vitro* biofilm formation and catheter occlusion.

### NAC displayed significant inhibition of clinical *P. mirabilis* biofilm formation and disruption of mature biofilms

**Figure 5:**
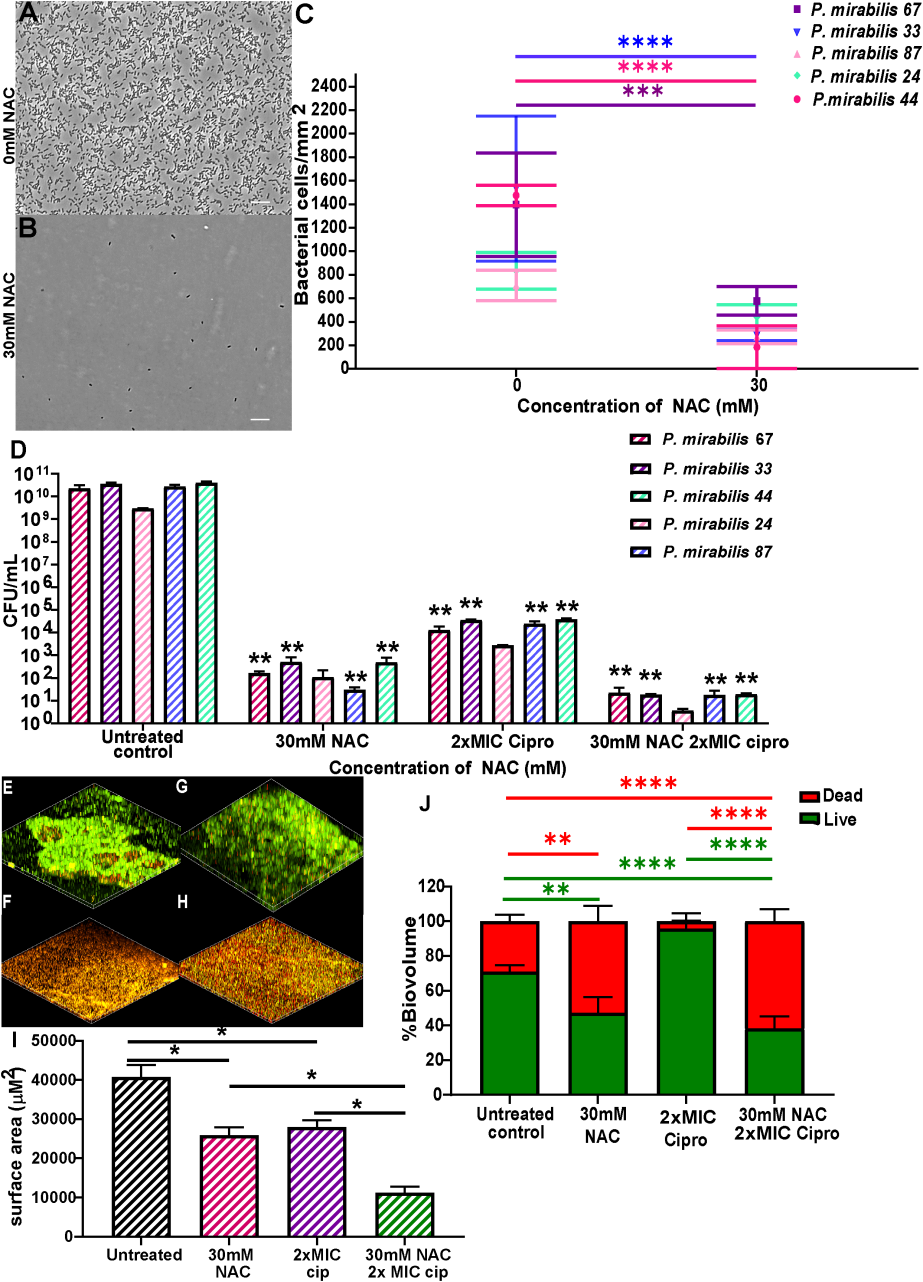
NAC prevented *P. mirabilis* adhesion to substratum and disrupted preformed biofilms *in vitro*. NAC significantly reduced initial adhesion of *P. mirabilis* isolates to substratum **(A-C)** compared to its untreated control. **(A, B)** show a representative image of the visual difference in adhesion as displayed by *P. mirabilis* 67. Scale bar: 10 μM **(D)** Significant disruption of 48 h biofilms and reduction in viable bacteria was recorded when treated with different concentrations of NAC. **p<0.01 relative to untreated controls. **(E-H)** Confocal images of biofilms stained with live/dead stain showing differences in biofilm architecture and level of disruption when treated with NAC and NAC + ciprofloxacin. **(E)** untreated control of *P. mirabilis* 67, **(F)** 30 mM NAC, **(G)** 6 μg/mL ciprofloxacin, **(H)** 30 mM NAC + 6 μg/mL ciprofloxacin **(I)** Quantification of surface area covered by biofilms under each treatment condition. **(J)** Quantification of live/dead biomass under each treatment condition. Sidak’s multiple comparisons testing was used for data in **(C)** and Tukey’s multiple comparison testing was used for the remaining statistical analyses reported. Unless otherwise stated, *p<0.05, **p<0.01, ***p<0.001, ****p<0.0001. Data represents the mean ± SD of n = 3 biological replicates.

NAC significantly prevented biofilm formation and disrupted mature biofilms in all five clinical isolates used in this study, thus showing broad spectrum antibiofilm activity against clinical isolates. Initial adhesion of all tested isolates was significantly inhibited (**Figures 5A–C**), with bacterial counts reduced from 800-2000 bacterial cells/mm^2^ to 0-600 bacterial cells/mm^2^ in the presence of 30 mM NAC. NAC also efficiently disrupted 48 h biofilms, alone and in combination with ciprofloxacin, the antibiotic of choice in combination therapy against *P. mirabilis* biofilm infections (See **Supplementary Figure 4** for concentration dependent effect of NAC). An >8log_10_ decrease in viable bacteria was observed across all five strains when treated with a combination of 30 mM NAC and 2xMIC ciprofloxacin (**Figure 5D**). This is a significant (p<0.01) 3-4log_10_ difference in colony counts when compared to treatment with 2xMIC ciprofloxacin. Slight differences in viable bacteria were present between NAC only and NAC+ ciprofloxacin treatment. The combination treatment not only resulted in enhanced killing of biofilm bacteria but also significantly disrupted the biofilm matrix, with a highly significant (p<0.0001) 40% increase in dead biofilm biovolume when treated with NAC alone or in combination with ciprofloxacin (**Figures 5E–J**). A variable quantity of dead biomass was obtained in untreated controls, possibly due to the 24 h incubation in 1 x PBS. **Figures 5F, H** show the clear reduction in live biovolume in NAC and combination-treated biofilms compared to their untreated and ciprofloxacin treated controls (**Figures 5E, G**, respectively).

### NAC hindered bacterial migration through catheter segments

**Figure 6:**
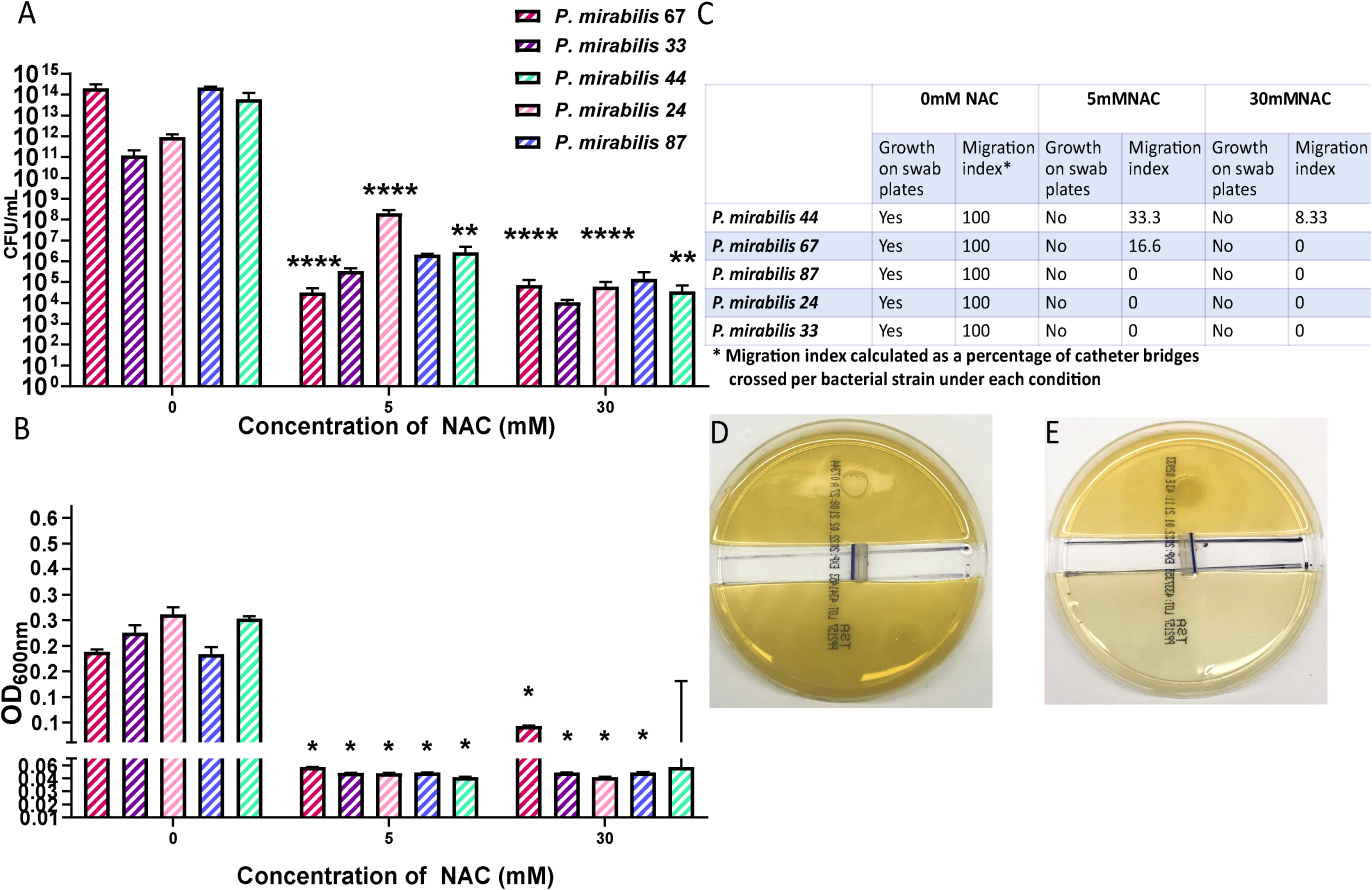
NAC prevented bacterial migration through catheter tubing. **(A)** Viable bacterial count obtained from catheter “bridges” that were scraped, sonicated, and plated on TSA plates for bacterial enumeration. **(B)** Growth as determined by OD, from a 1x1 cm^2^ square of agar on the uninoculated side, to measure bacterial presence and migration. **(C)** Summary of *P. mirabilis* migration through catheter bridges in presence of NAC. **(D, E)** Representative images of *P. mirabilis* 67 migration and growth in 0-, and 30 mM NAC, respectively. Tukey’s multiple comparisons testing used for statistical analysis. *p<0.05, **p<0.01, ****p<0.0001, compared to untreated controls. Data represent the mean of ± SD of n = 4 biological replicates, with three technical replicates in each experiment.

To investigate whether the inhibition in bacterial adhesion seen in **Figure 5C** is mirrored in the catheter lumen, a bacterial migration assay was performed to quantify bacterial adherence and migration through a catheter in the presence of NAC. A significant reduction in the amount bacteria found within the catheter segment was observed in the presence of NAC. **Figure 6A** shows the significant (p<0.0001) 9log_10_ reduction in CFU/mL observed for *P. mirabilis* 67, and the significant 8log_10_ reduction in CFU/mL for *P. mirabilis* 87 and *P. mirabilis* 44 (p<0.0001 and p<0.01 respectively). A non-significant but equally large reduction in adhered bacteria was also noted for *P. mirabilis* 33 and *P. mirabilis* 24. The reduction in bacterial attachment in the catheter segments at 5 mM and 30 mM NAC were similar. No growth was observed on the uninoculated half of the agar in the NAC treated catheter segments (**Figures 6B, C**), compared to respective untreated controls, where growth was present on all swab plates done from the uninoculated agar, and growth reached an OD_600nm_ of almost 0.4. Three out of five strains recorded no migration at all in the NAC treated catheter segments. However, *P. mirabilis* 44 and 67 demonstrated migration of 33% and 16%, respectively, in the 30 mM NAC treated catheter segments. Despite this migration, the strains still had a significantly smaller load of adhered bacteria compared to untreated controls.

### Prevention of catheter encrustation by *P. mirabilis* in the presence of NAC

**Figure 7:**
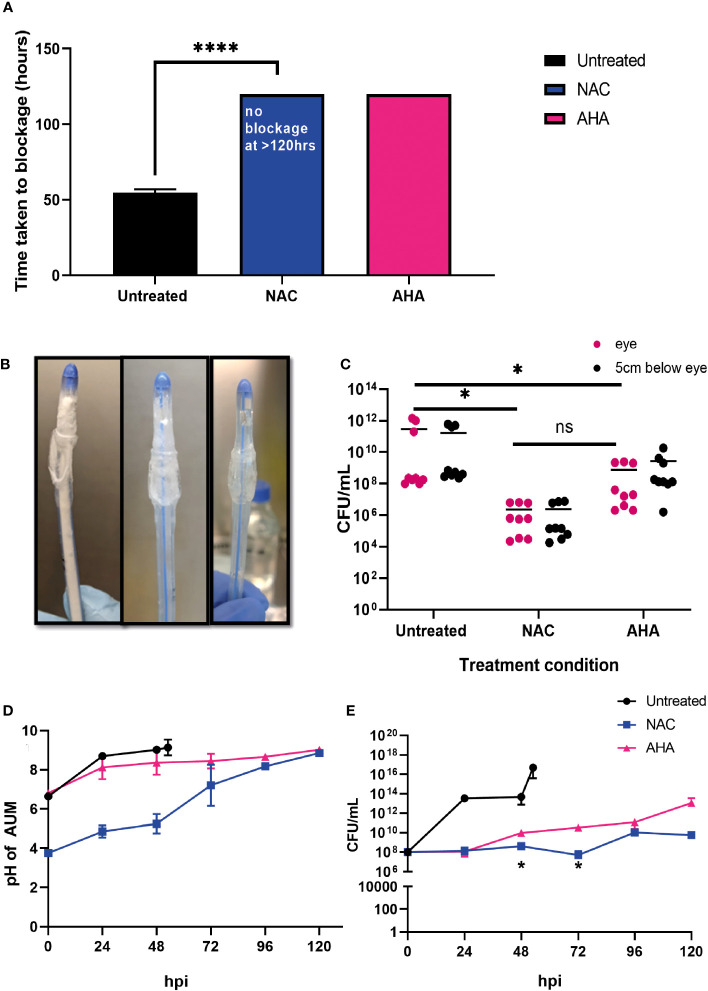
NAC prevented catheter encrustation by *P. mirabilis in vitro*. **(A)** Comparison of time taken to cause catheter occlusion by *P. mirabilis* 67 in the presence of 10 mM AHA and 30 mM NAC. **(B)** Visual comparison of Untreated, NAC and AHA treated catheters respectively at time of occlusion or experimental endpoint. **(C)** Enumeration of viable bacteria present at time of catheter harvest (corresponding to time of occlusion or experimental endpoint), in catheter biofilms. **(D, E)** Measurement of artificial urine pH and bacterial counts in artificial urine, respectively, in the bladder, taken at 24 h intervals until time of catheter occlusion or experimental endpoint. Dunnett’s T3 multiple comparison’s testing was performed for statistical analysis. *p<0.05, ****p<0.0001. Data represent the mean ± SD of n = 3 biological replicates. ns, not significant.

To determine if the combined antibiofilm property and anti-urease effect of NAC translated into preventing catheter encrustation *in vitro*, a catheterised glass bladder model (**Figure 1**) was inoculated with *P. mirabilis* 67 and ‘time to blockage’ was quantified in the presence of 30 mM NAC. While the untreated catheters were blocked within 50 h, no blockage had occurred in the presence of 30 mM NAC by the conclusion of the experiment (120 h) (p<0.0001) (**Figure 7A**). This was comparable to the results obtained from catheters treated with AHA in the same experiment. Visually, the untreated catheter was occluded with struvite and apatite crystallised deposits within the biofilm, whereas no deposits were present in the NAC and AHA treated catheters (**Figure 7B**). The bacterial loads recovered from the NAC treated catheters were significantly lower (p<0.05) at the 120 h timepoint than that from untreated catheters at the 50 h timepoint (a 4log_10_ decrease in CFU/mL) (**Figure 7C**). The urinary pH only surpassed the neutral pH mark at around the 96 h timepoint in the presence of NAC (**Figure 7D**), with bacteria in the bladder below 10^8^ CFU/mL in the same. The lower pH observed in AUM containing NAC is due to the intrinsic acidity of NAC. This is significantly lower than the loads recovered from the untreated control (p<0.05), which reached a 10^16^ CFU/mL at the point of blockage.

### Treatment with NAC significantly influenced the elemental profiles of catheter biofilms

**Figure 8:**
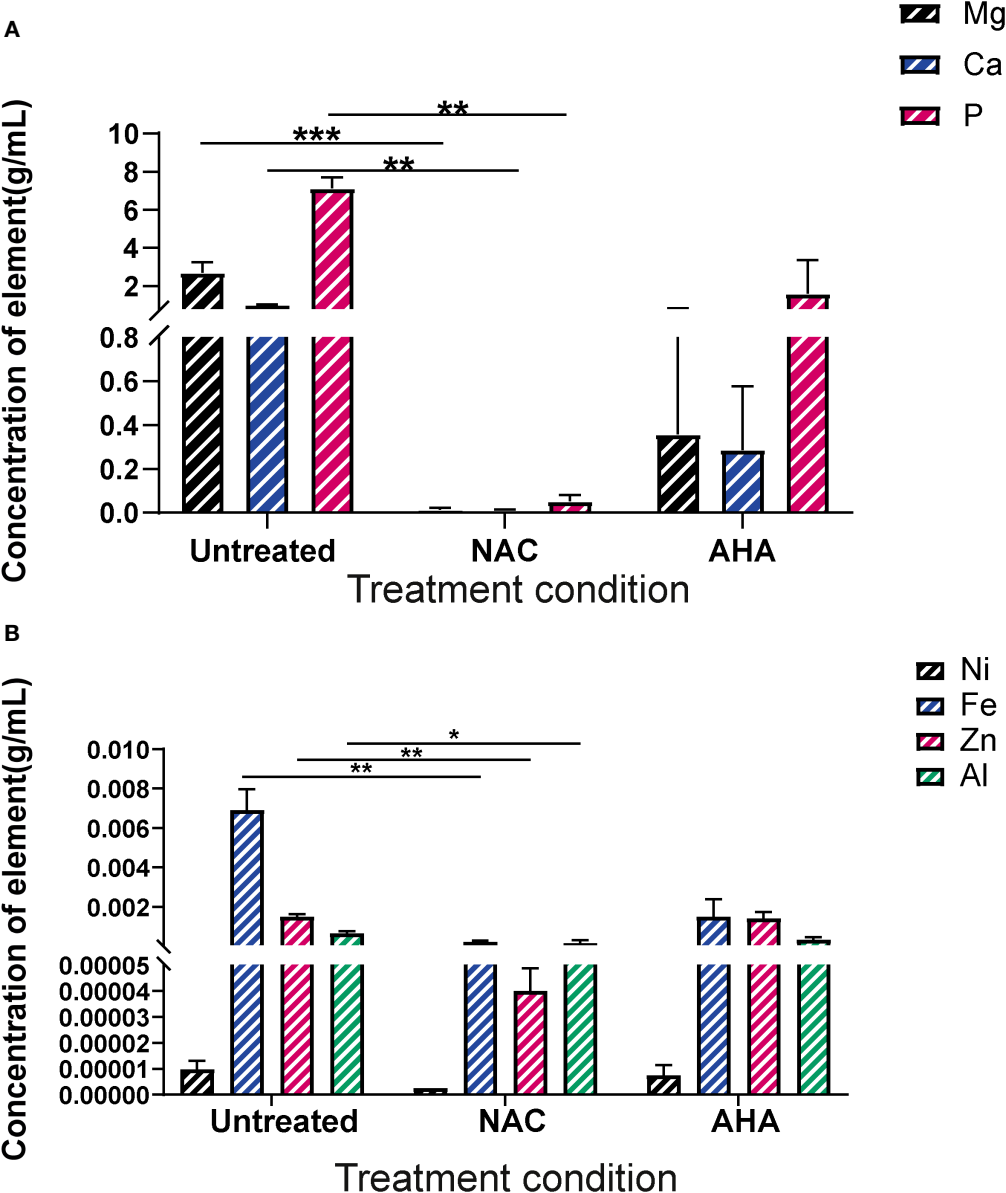
Elemental profiles of biofilms formed in catheters *in vitro* under different treatment conditions. Elemental analysis of the biofilms formed in the eye of catheters harvested at occlusion or at experimental endpoint was profiled using ICP-MS. **(A)** Concentration of magnesium, calcium and phosphorus present in crystalline biofilms formed in catheter eye. **(B)** Concentration of Nickel, Iron, Zinc and Aluminium present in crystalline biofilms formed in catheter eye. Dunnett’s T3 multiple comparisons test performed for statistical analysis. *p<0.05, **p<0.01, ***p<0.001. Data represent the mean ± SD of n = 3 biological replicates.

Biofilms were recovered from the eye of harvested catheters and subjected to ICP-MS to elucidate the effect of NAC on the elemental profiles of biofilms and deposits found in the catheter at time of harvest. In presence of NAC, magnesium and calcium levels were significantly lower when compared to much higher concentrations of 2.7 and 1 g/mL, respectively in the untreated control (p<0.01) (**Figure 8A**). Mg^2+^ and Ca^2+^ levels in the NAC treated catheters were almost 100 times less than concentrations found in AHA catheters (0.36 and 0.29 g/mL respectively). A similar trend was observed in Phosphorus concentrations, where only 0.05 g/mL was detected in the NAC treated catheters, compared to 7.12 in the untreated controls, a significant difference (p<0.01). The concentrations of other elements such as iron and zinc were also significantly lower in the NAC-treated catheters, where 0.0002 g/mL and 0.00004 g/mL, respectively, were recorded (p<0.01) (**Figure 8B**). This was a >100-fold decrease compared to the untreated control, where Fe and Zn were found at concentrations of 0.007 and 0.001 g/mL, respectively. Fe and Zn in the AHA treated catheters were found at levels almost comparable to the untreated control, at concentrations of 0.0014 g/mL for both, thus displaying no significant difference.

### Inflammatory response of cells to urease and *P. mirabilis* infection was subdued with NAC treatment

**Figure 9:**
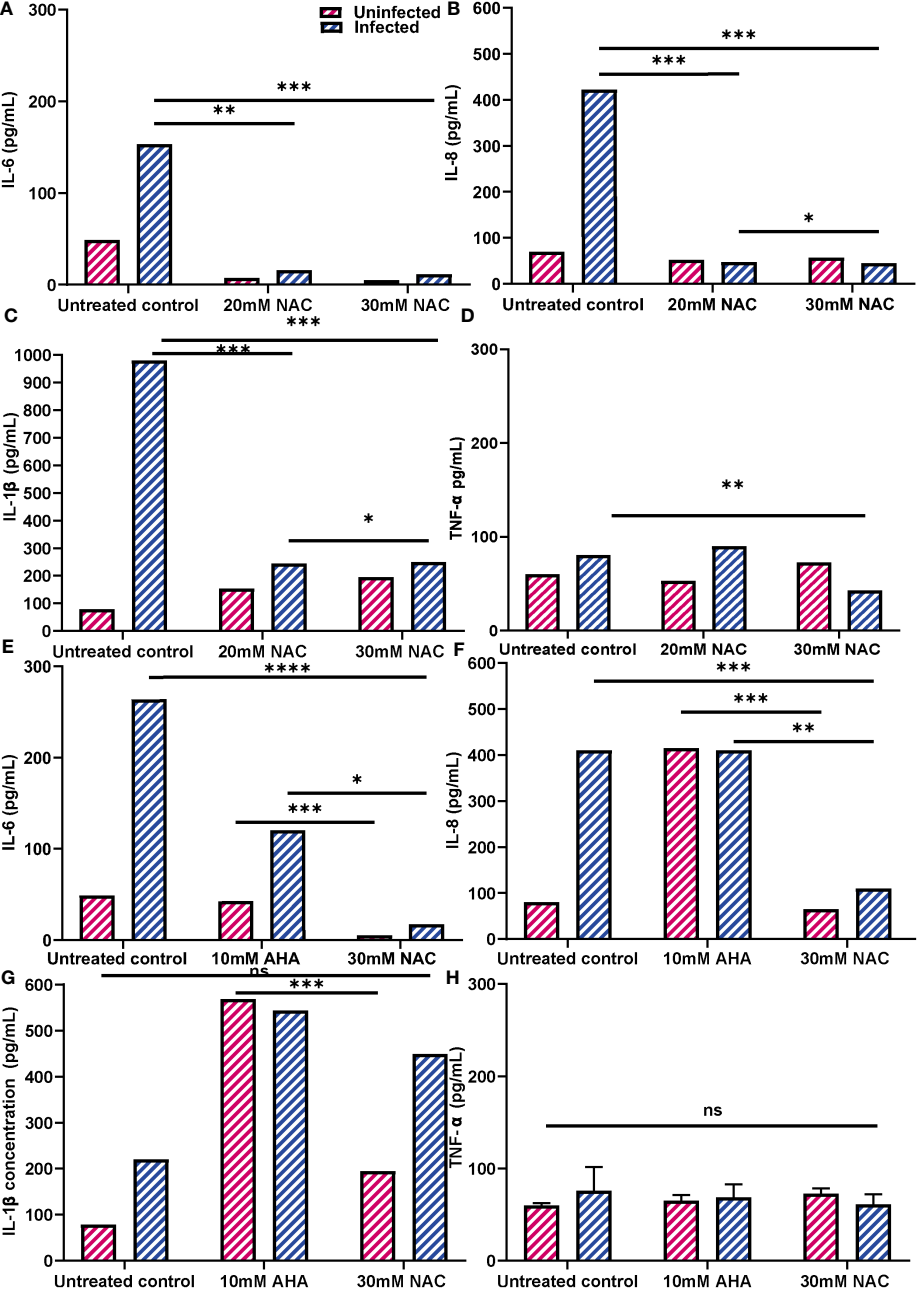
Inflammatory profiles of 5637 BECs to *P. mirabilis* infection or JBU in presence of NAC. **(A-D)** IL-6, IL-8, IL-1b and TNF-α production in response to *P. mirabilis* infection in the presence of varying concentrations of NAC (in blue). Uninfected conditions are in pink. BECs were infected with *P. mirabilis* and incubated for 2 h prior to supernatant collection for cytokine analysis. **(E-H)** IL-6, IL-8, IL-1b and TNF-α production in response to JBU in presence of AHA and MBIC concentrations of NAC. The difference in cytokine production between AHA and NAC treatments were also observed. Dunnett’s T3 multiple comparisons test was performed for statistical analysis. *p<0.05, **p<0.01, ***p<0.001, ****p<0.0001. Data represent the mean ± SD of n = 3 biological replicates. ns, not significant.

The effect of NAC treatment on cytokine secretion when BECs were infected with *P. mirabilis* was quantified using ELISAs (**Figures 9A–D**). As expected, *P. mirabilis* infection resulted in very high levels of IL-6 and IL-8, (153 and 422 pg/mL, respectively), over 3-4 times higher than their respective baseline levels. The IL-1b response was the strongest, with levels of up to 960 pg/mL observed (**Figure 9C**). However, in presence of NAC, IL-6, IL-8 and IL-1b levels fell significantly, more than 10fold below the infected control levels, which were 11, 44 and 250 pg/mL, respectively, (p<0.001, p<0.0001). No concentration-dependent effect of NAC was noted, except in the case of IL-1b, where a slightly significant difference was found between 20 mM and 30 mM (p<0.05) (**Figure 9D**). Interestingly, there was a minor upregulation of TNF-α production in response to infection, however, when treated with 30 mM NAC, TNF-α production to infection decreased (p<0.01).

Additionally, urease was found to be highly stimulatory, stimulating IL-6 and IL-8 secretion by >100 pg/mL and 400 pg/mL, respectively, (**Figures 9E, F**). In comparison, IL-1b and TNF-α responses to urease were not as strong (**Figures 9G, H**). AHA was also highly inflammatory, resulting in IL-6, IL-8 and IL-1b levels of 60 pg/mL, 400 pg/mL and 580 pg/mL, respectively. More interestingly, when BECs exposed to urease were treated with AHA, the IL-6 and IL-8 concentrations were almost identical to those obtained with BEC stimulation of urease and AHA independently. However, when urease- exposed cells were treated with 30 mM NAC, cells produced much lower levels of IL-6 and IL-8 (<20 pg/mL and 100 pg/mL, respectively) (p<0.05, p<0.01). By comparison, NAC treatment did not induce an inflammatory response, with concentrations of IL-6 and IL-8 being over five times lower than those recorded with AHA (p<0.001). While NAC alone did not stimulate a strong IL-1b response, when NAC was combined with urease, IL-1b levels were at 460 pg/mL (**Figure 9G**). The TNF-α response by BECs to urease, AHA and NAC, independently and in combination, remained relatively the same as the unstimulated controls (**Figure 9H**). In all cases, the uninfected controls provide a comparison to cytokine production by NAC alone.

### NAC reduced the level of BEC invasion by *P. mirabilis*

**Figure 10:**
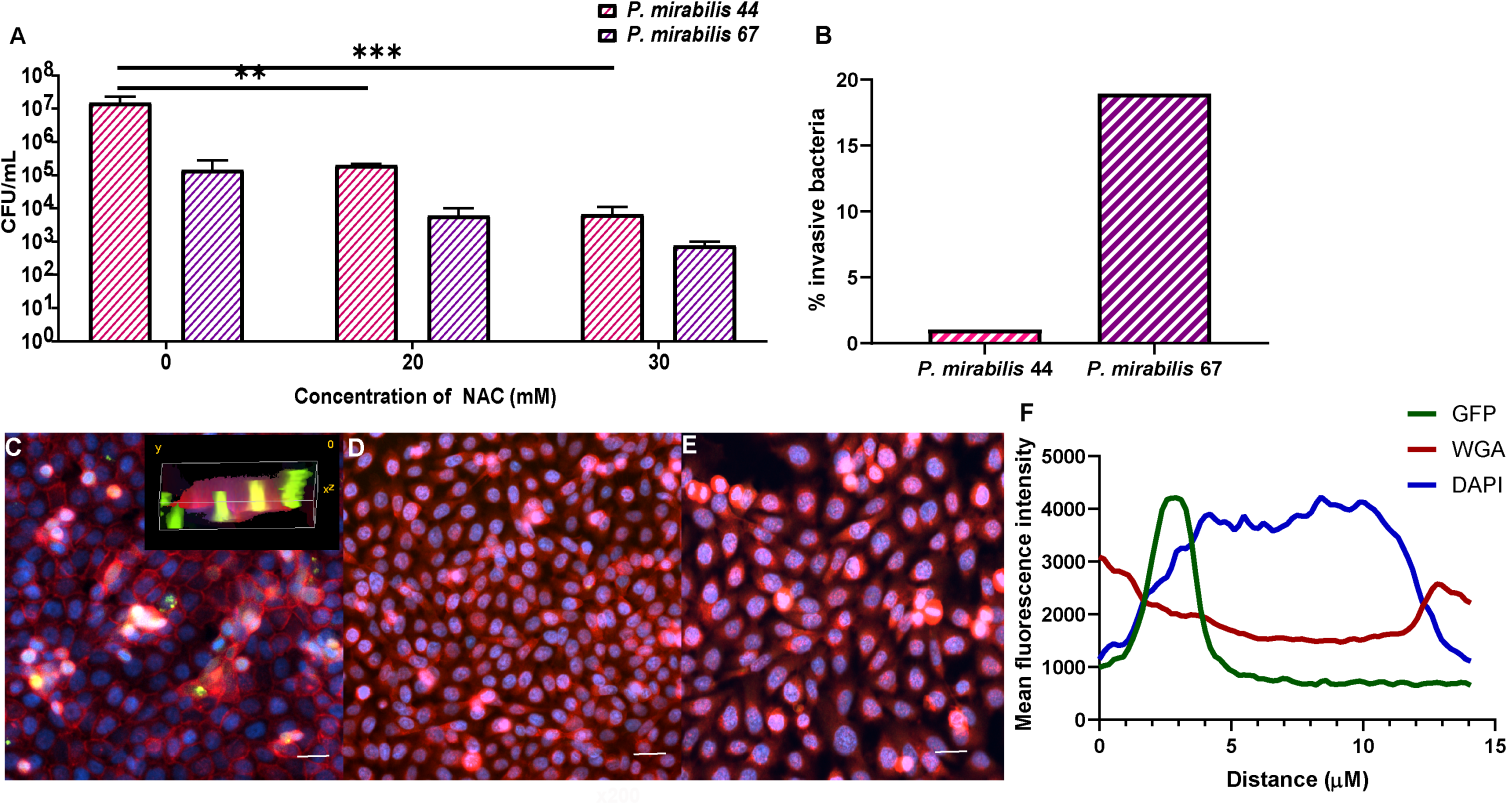
Treatment with NAC significantly reduced bacterial invasion of BECs. **(A)** BECs infected with either *P. mirabilis* 44 or *P. mirabilis* 67 for 2 h displayed a significant reduction in internalised bacteria in the presence of NAC, in a concentration dependent manner. **(B)** Percentage of infecting bacteria internalised by cells. **(C)** Visualisation of invasion of BECs by fuGFPb-tagged *P. mirabilis* 44 in the absence of NAC, with the inset image depicting a 3-D visualisation of invaded BECs. **(D, E)** Invasion of BECs in the presence of 20mM and 30mM NAC, respectively Scale bar: 50 μM. **(F)** Mean fluorescence intensity of different channels through an infected BEC. Tukey’s multiple comparisons test was used for statistical analysis. **p<0.01, ***p<0.001. For **(A)**, data represent the mean ± SD of n = 3 biological replicates.

NAC treatment reduced *P. mirabilis*-mediated invasion of BECs. The intracellular bacterial burden 2 h post infection was 2-3log_10_ less in the presence of NAC for both *P. mirabilis* strains tested, compared to their respective untreated controls (**Figure 10A**). For *P. mirabilis* 44¸ the untreated control had an intracellular burden of 10^7^ CFU/mL, whereas after treatment with 30 mM NAC, only 10^4^ CFU/mL were recovered (p<0.001). For *P. mirabilis* 67, the difference between treated and untreated conditions was non-significant, however there was still a 2log_10_ reduction in intracellular bacteria in the presence of 30mM NAC. As shown in **Figure 10B**, *P. mirabilis* 67 was the more invasive strain. Difference in invasion was visualised using a fuGFPb-tagged *P. mirabilis* 44, where in the untreated control, bacterial invasion was clearly visible (**Figure 10C**), however in the presence of 20- and 30 mM NAC, no bacterial invasion was visible (**Figures 10D, E**). The plot profiles depict the spike in GFP coinciding with a decrease in WGA and increase in DAPI, as a drawn line moves through an invaded cell, thus suggesting internalisation of bacteria within and not outside the BEC (**Figure 10F**).

## Discussion

In this study, we have shown that NAC is a potent inhibitor of bacterial urease at low concentrations (**Figure 2**). It is important to note that the anti-ureolytic activity of NAC does not rely on having substance at its normally acidic pH, with lower concentrations existing at a more neutral pH (**Supplementary Table 3**). This is relevant as highly acidic solutions are damaging to the bladder. Additionally, NAC enhances biofilm disruption (**Figure 5**) and interestingly, subdues the inflammatory response of BECs to infection (**Figure 9**). Using a substance such as NAC is a powerful approach to combat antibiotic resistance as it targets urease, a factor that can enhance intrinsic resistance; and limits inflammatory mediated damage to bladder epithelial cells. Put together, this treatment can facilitate improved outcomes with antibiotic therapy.

The presence of a free thiol group is pivotal for NAC activity and thiols are restricted to cysteine and closely related molecules (Sissons and Yakub, 2000). Studies have investigated the anti-urease efficacy of molecules containing free thiols (Díaz-Sánchez et al., 2016; Milo et al., 2021). L-cysteine and cysteine both show strong anti-ureolytic activity (Sissons and Yakub, 2000; Svane et al., 2020), while GSH, (synthesised from NAC), showed no anti-ureolytic activity (Sissons and Yakub, 2000). Additionally, preliminary findings showed that when *P. mirabilis* was treated with glutathione disulphide (GSSG), the oxidised version of GSH, no anti-ureolytic activity was evident. Evidence from the current study indicates NAC potentially binds to the active site in a competitive manner, since the increased Michaelis-Menten constant (Km) denotes a reduced affinity for urea in the presence of NAC (**Figure 3A**). Moreover, structural modulation of urease by NAC is evident, as shown by the CD spectra data (**Figure 4**). Inhibiting urease in the presence of urea is key to limiting *P. mirabilis* pathogenesis as urease is a urea-inducible enzyme and is easily induced in urine where urea concentrations can reach 400 mM (Armbruster et al., 2018).However as the Michaelis-Menten model only works under highly controlled conditions, results must be interpreted with care (Milo et al., 2021). Future targeted studies aim to determine target of NAC on key urease gene expression (if any) and which subunit of the enzyme structure is modified in the presence of NAC. Inhibition of urease gene expression by NAC treatment in the presence of urea would strongly diminish the virulence of *P. mirabilis*, and its ability to obstruct catheters. Studies are also needed to investigate the level of NAC uptake by *P. mirabilis* during treatment, to quantify effect on cytoplasmic urease and its production. This study did not quantify the amount of enzyme itself present after NAC treatment, which is an important factor to consider in future studies, both at the transcriptional and translational levels in the bacteria. The presence of thiols is hypothesised to reduce urease activity by creating a falsely alkaline environment arising from protein hydrolysis, thus repressing the need for urease activity (Sriwanthana and Mobley, 1993).

This study demonstrated the effects of NAC in various stages of biofilm formation, including initial adhesion to disruption of mature biofilms when combined with ciprofloxacin (**Figure 4**). Our results do corroborate the previous work of Abdel-Beky et al. (2017) (El-Feky et al., 2009). However, the study used a concentration over 100 times more than what we tested and did not describe cellular effects of NAC treatment. Moreover, we have also shown the broad spectrum antibiofilm activity of NAC against numerous clinical *P. mirabilis*, including inhibition of biofilm formation on catheters. The antibiofilm activity of NAC is hypothesised to stem from its thiol (-SH) region interacting with bacterial cell wall proteins and interrupting bacterial cysteine utilisation (Blasi et al., 2016). Investigating the effect of NAC on multispecies biofilms implicated in CAUTIs including *P. aeruginosa* and *P. mirabilis* will also be an important future avenue, especially given that the bladder is not sterile and most CAUTIs are polymicrobial, given its exposure to unsterile urine.

No catheter blockage, and negligible struvite and hydroxyapatite deposits were recorded in the presence of NAC after 120 h (**Figure 7**). Blockage in the untreated control occurred 52 h post infection, which was expected as *P. mirabilis* catheter infection takes ~50 h to block silicon catheters (Jacobsen and Shirtliff, 2011). However, the stark difference in elemental profiles of catheters treated with AHA vs NAC was unexpected (**Figure 8**). The depletion of magnesium and calcium in the NAC treated catheters mirrors the absence of any calcium struvite and hydroxyapatite deposits, despite the urinary pH slowly climbing in the latter stages of infection. While the cause of this delayed pH rise is unclear, it could be due to NAC degradation or the presence of residual bacteria in the bladder. This might also correlate with the slight increase in bacterial numbers at the latter timepoints, given that NAC is bacteriostatic on *P. mirabilis* (**Supplementary Figure 2C**). The lack of crystalline deposits, combined with the decrease in viable biofilm bacteria suggest that NAC, could be an excellent candidate for bladder washout or antimicrobial coating at any stage of infection. The migration studies (**Figure 6**) have shown that the presence of NAC in the catheter sections at MBICs does prevent bacterial swarming and colonisation of the catheter, including by any surviving populations. These results provide a strong impetus for future immobilisation studies onto silicon and hydrogel catheters. NAC can be immobilised onto other abiotic surfaces such as PVC while sustaining significant antibiofilm activity (Costa et al., 2017; Yang et al., 2020), and is proof that catheter surfaces can be coated similarly. It is theorised that NAC coatings prevent bacterial adhesion to substratum using various methods such as decreasing surface hydrophilicity or interrupting bacterial protein adsorption to surfaces (Costa et al., 2017).When taken together, these twin properties of NAC (antibiofilm and anti-urease/crystal formation) significantly advance its’ potential use in preventing catheter encrustation.

For the first time, our study has demonstrated the highly inflammatory response of bladder cells to urease, with IL-6 and IL-8 production dramatically enhanced. IL-6 and IL-8 are the main cytokines found highly elevated in patients with UTIs (Ko et al., 1993; Ching et al., 2018). IL-6 promotes the acute phase febrile response, neutrophil production, and B-cell activation, while IL-8 acts as a chemoattractant for neutrophils at the site of infection (Fünfstück et al., 2001). In the presence of NAC, the strong inflammatory response to urease and to infection with *P. mirabilis* was markedly subdued. If replicated *in vivo* (work currently under progress), this could potentially aid in preventing enhanced tissue damage to the epithelium. Urease has been described as a moonlighting protein given its multiple biologically relevant non-enzymatic properties. Studies have previously shown the effect of urease on HEK-293 kidney cells, where IL-1b and TNF-a production increased in a dose-dependent manner (Grahl et al., 2021). We have extended this in the finding of strong IL-1b secretion by bladder cells in the presence of not only urease alone, but also AHA, and *P. mirabilis* infection (**Figures 9C, G**). While it is believed that the crystalline deposits resulting from urease activity primarily induce the IL-1b response (Seo et al., 2015; Armbruster et al., 2017), the potent IL-1b response to AHA was unexpected. IL-1b is associated with severe acute cystitis in the presence of high bacterial burdens (Butler et al., 2022), hence treatment induced inflammation should be limited.

This study demonstrated that NAC is not cytotoxic to BECs at optimal concentrations using ISO standard methods (**Supplementary Figure 5**), as no cell death was recorded at 24 h. Additionally, NAC was anti-inflammatory and did not induce IL-6, IL-8 and IL-1b production (**Figure 9**), thus providing evidence of its safety as a UTI treatment. While the alternative AHA was not visibly cytotoxic (**Supplementary Figure 5**), it stimulated over four times more IL-6 and IL-8 than NAC (**Figures 9A, B**). AHA’s highly inflammatory nature coupled with severe side effects (Kosikowska and Berlicki, 2011), has promoted extensive research efforts to identify alternate urease inhibitors. The FDA-approved dose of NAC for use in humans is almost 200 times higher than the MIC needed for treating uropathogens according to this study and previously published studies (Manoharan et al., 2021). It equates to 10.5 g in a 70-kg human (approximately 6-7 M). The fact that only a fraction of this amount is needed for UTI provides further proof of the utility of NAC for use in the urinary tract in a site-specific manner. Additionally, NAC has previously been used in patients to treat other conditions such as mucus production in cystic fibrosis patients, as an anti-inflammatory medication, as well as an antiviral agent for example (Mokhtari et al., 2017). In many of these treatment regimes NAC is used as cyclical therapy, with an initial loading dose, followed by multiple rounds of maintenance doses during treatment (Tenório et al., 2021). This further eliminates the concern of the potential translation of invitro findings to clinical applications. Currently, the most common side effects of NAC treatment include diarrhoea and bloating (Rhodes and Braakhuis, 2017). Additionally, side effects are also concentration dependent, with adverse effects only seen at much higher concentration than required to treat CAUTIs. Further investigations are required to establish the concentrations of NAC achievable in the bladder, as well as the risks associated with extended treatment of UTIs with NAC.

The pro-inflammatory effect of urease in the context of *H. pylori* urease (HPU) has been studied extensively on cells of a gastric origin, where neutrophils are overactivated by HPU due to the over expression of IL-1b and TNF-a in a dose-dependent manner. This in turn has been shown to significantly contribute to gastric carcinogenesis associated with *H. pylori* infections (de Jesus Souza et al., 2019). Given that IL-6 and IL-8 are highly expressed in response to UPEC infections and are associated with increased intracellular bacterial colony formation (Fünfstück et al., 2001), combined with the fact that urease is critical to *P. mirabilis* in bladder colonisation (Armbruster et al., 2017), our findings provide a springboard to further investigations into whether urease promotes tissue damage that facilitates bacterial invasion and colonisation.

This study has comprehensively investigated the effects of NAC on *P. mirabilis* catheter encrustation and biofilm infection using a multi-disciplinary approach. Inhibiting urease activity with NAC therapy significantly impeded *P. mirabilis* crystalline biofilm formation and catheter occlusion *in vitro*. This is a pivotal finding as biofilm disruption and eradication is the goal in the context of CA-UTIs. Moreover, the anti-inflammatory effect of NAC on BECs in response to urease as well as *P. mirabilis* infection, combined with the finding that NAC prevents BEC invasion *in vitro* by *P. mirabilis* showed that NAC plays an important role on both sides of the host-pathogen axis. This warrants further investigation into whether NAC can limit subsequent tissue invasion and damage during infection *in vivo* in animal models.

Ultimately, further work to develop these findings on NAC treatment of *P. mirabilis* UTI infection will provide a strong basis for a way of preventing acute and chronic CA-UTIs in hospital settings and establish a paradigm shift in the way we approach biofilm infections in CA-UTIs.

## Data availability statement

The raw data supporting the conclusions of this article will be made available by the authors, without undue reservation.

## Author contributions

AM and TD conceptualised and designed the study. Methodology: AM, JF, TD, VA, and NK. AM performed formal analysis, investigation, and interpretation. AM, TD, and VA performed the experiments. Manuscript preparation: AM, writing—review and editing, AM, GW, EK, TG, JF, KM, VA, NK, JM, and TD; supervision, JM and TD; project administration, TD, JM, and JF. All authors contributed to the article and approved the submitted version.
