# Supplementary material for: N-acetylcysteine prevents catheter occlusion and inflammation in catheter associated-urinary tract infections by suppressing urease activity
Source: Front Cell Infect Microbiol. 2023 Oct 26;13:1216798. doi: 10.3389/fcimb.2023.1216798 (PMC10641931; doi:10.3389/fcimb.2023.1216798)
Supplement: Supplementary file 1 [file DataSheet_1.docx]

**Supplementary Figures**

**Supplementary Table 1. Source and antibiotic susceptibility profiles of all isolates used in this study.** RPAH- Royal Prince Alfred Hospital, CIP- Ciprofloxacin, TMZ-SMX- Trimethoprim-sulfamethoxazole, CRO- Ceftriaxone, NFN-nitrofurantoin. R- Resistant, S-Susceptible.

| Bacterial species | Source |  | Antibiotics | | |
| --- | --- | --- | --- | --- | --- |
|  |  | **CIP** | **TMZ-SMX** | **CRO** | **NFN** |
| *P. mirabilis* 67 | Urine - RPAH, Sydney, Australia | R | R | R | R |
| *P. mirabilis* 44 | Genital - RPAH, Sydney, Australia | S | S | S | R |
| *P. mirabilis* 33 | Urine- RPAH, Sydney, Australia | S | R | S | R |
| *P. mirabilis* 24 | Blood culture- RPAH, Sydney, Australia | S | S | S | R |
| *P. mirabilis* 87 | Urine - RPAH, Sydney, Australia | S | S | S | R |

**Supplementary Table 2 ELISA kits obtained from Abcam**

| ELISA kit | Abcam catalogue number |
| --- | --- |
| Human IL-6 | Ab46027 |
| Human IL-8 | Ab214030 |
| Human IL-1b | Ab214025 |
| Human TNF-a | Ab181421 |

**Supplementary Table 3 pH of NAC at different concentrations in PBS**

| **Concentration of NAC** | **pH** |
| --- | --- |
| 0.5 mM | 7.16 |
| 1 mM | 7.11 |
| 5 mM | 6.30 |
| 10mM | 3.59 |
| 30mM | 2.66 |

**
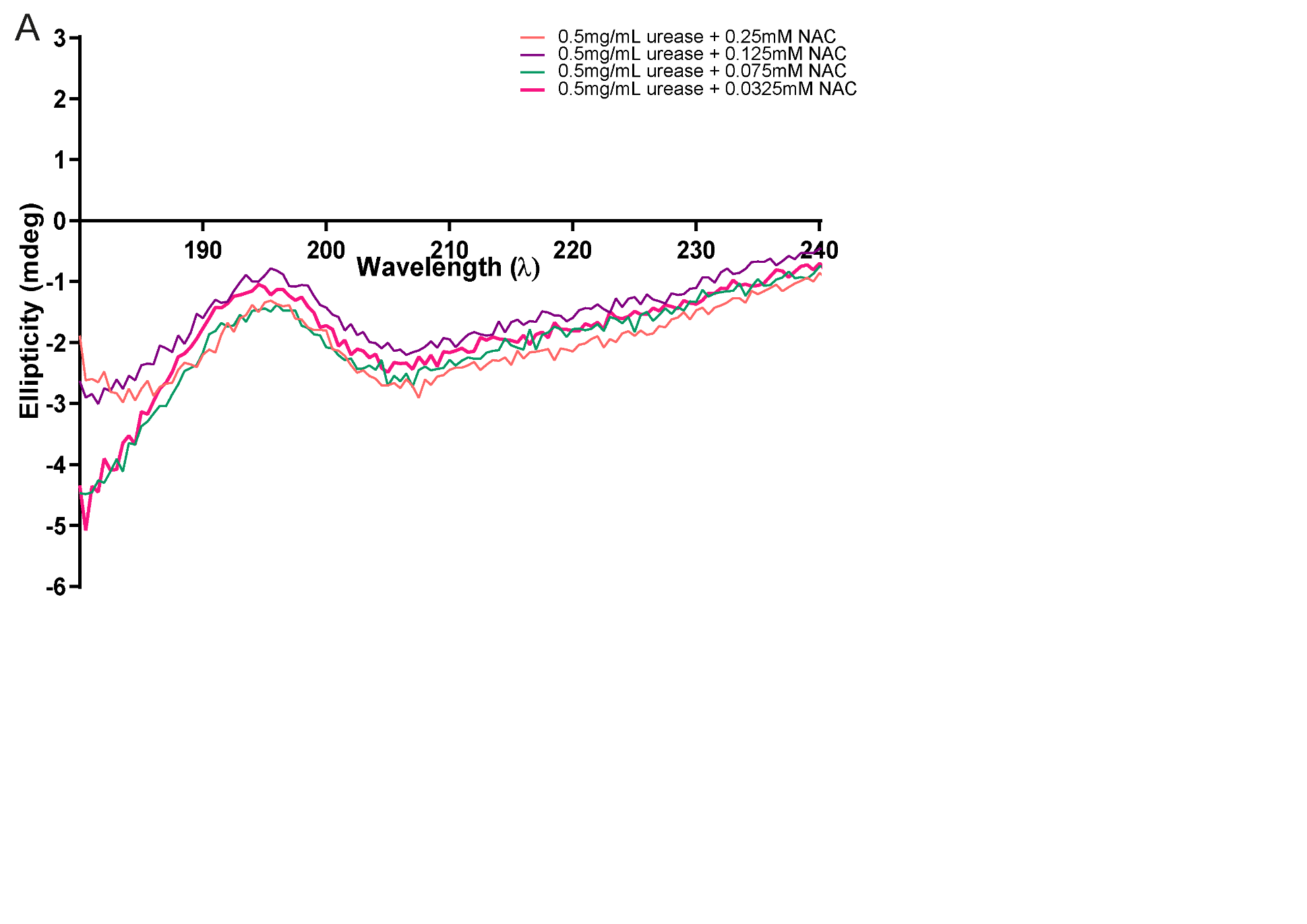
**

**Supplementary Figure 1 Circular dichroism spectra showing the concentration dependent effect of NAC on urease**

**A** The concentration dependent effect of NAC on the structure of 0.5 mg/mL urease as shown by the comparison of CD spectra.

**
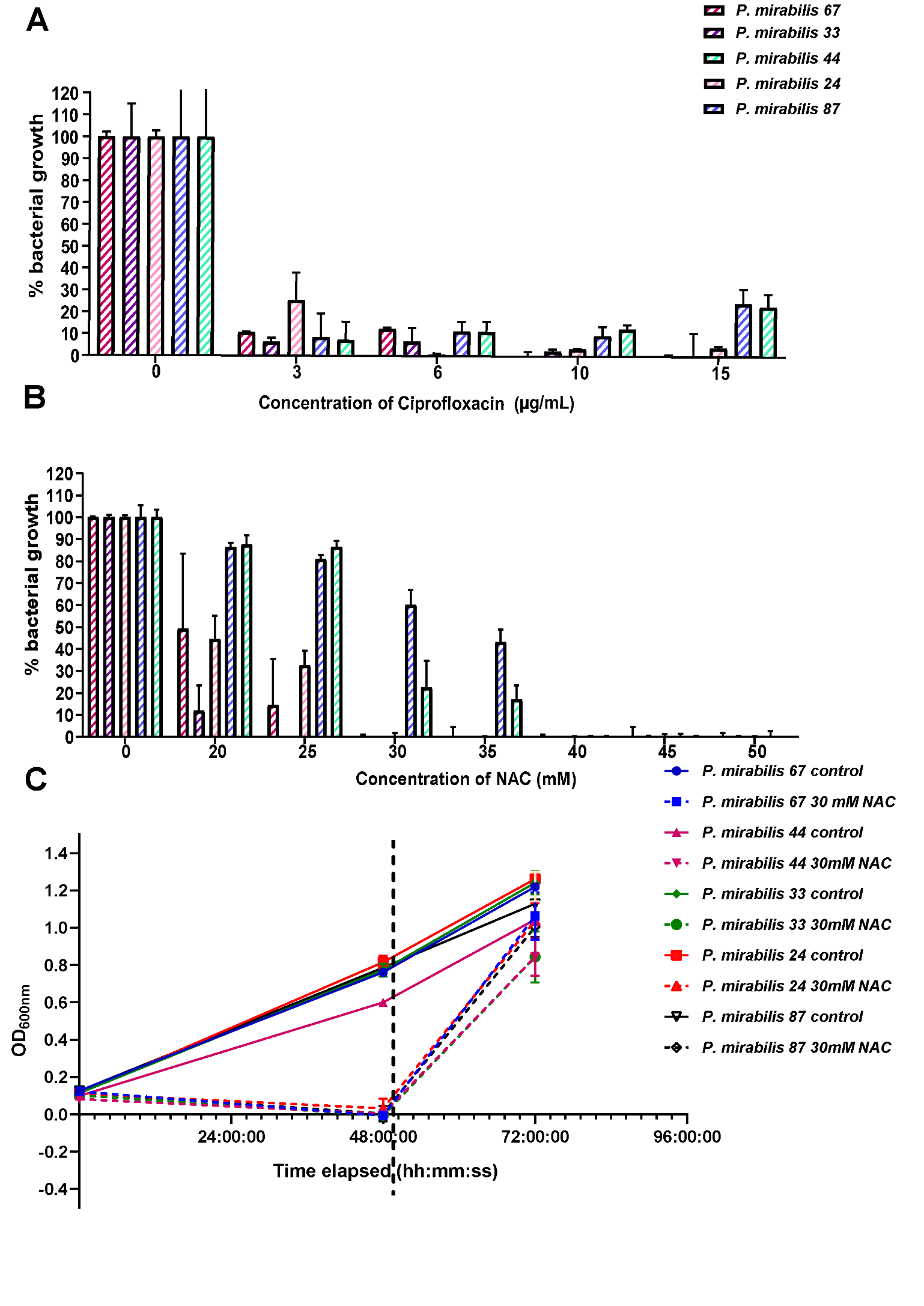
**

**Supplementary Figure 2 Determination of NAC and ciprofloxacin MICs in all tested clinical *P. mirabilis* strains and establishment of bacteriostatic effect of NAC .**

**A** Ciprofloxacin MICs were determined for all five *P. mirabilis* clinical isolates used in this study. **B** NAC MICs were determined for all five *P. mirabilis* clinical isolates used in this study.

**
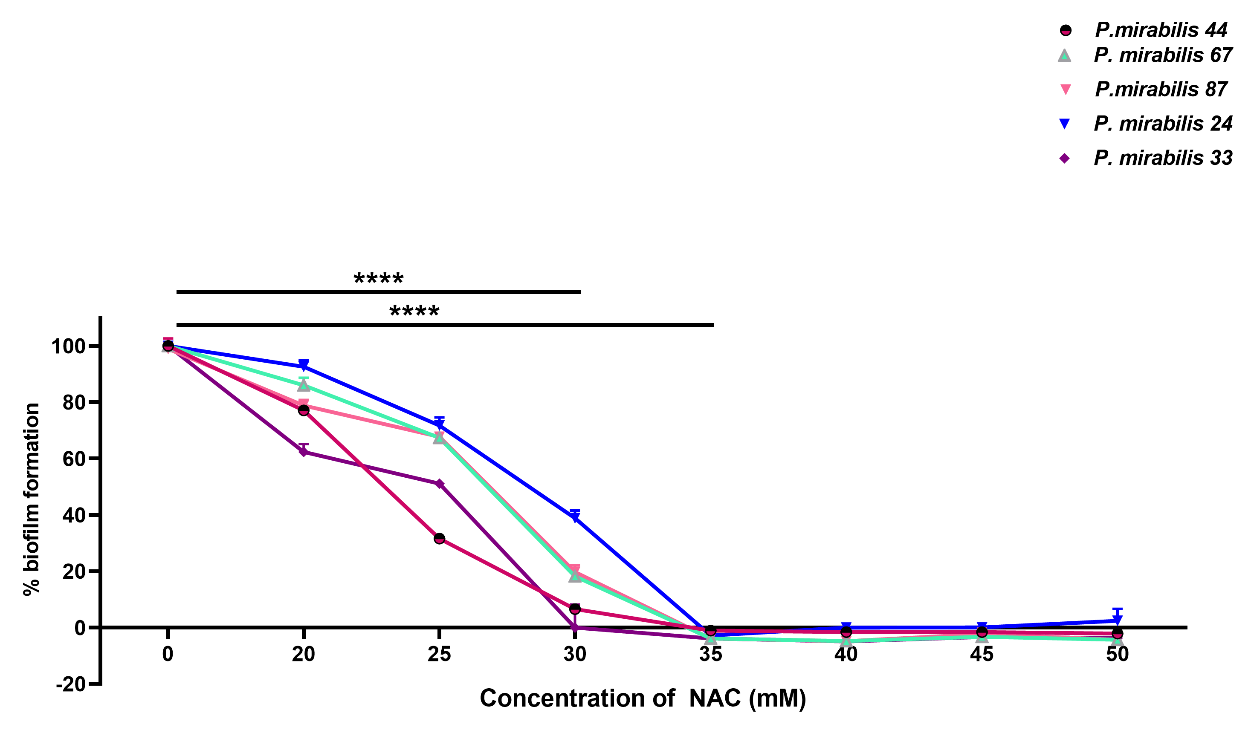
**

A

**Supplementary Figure 3 Determination of the minimum biofilm inhibitory concentration (MBIC) of NAC for all tested clinical *P. mirabilis* strains**

**A** The MBIC of NAC for all clinical strains of *P. mirabilis* used in this study was determined on 48 h old biofilms. Tukey’s multiple comparison testing was used for statistical analysis. ****=p<0.0001. Data represent the mean ± SD of n = 4 biological replicates.


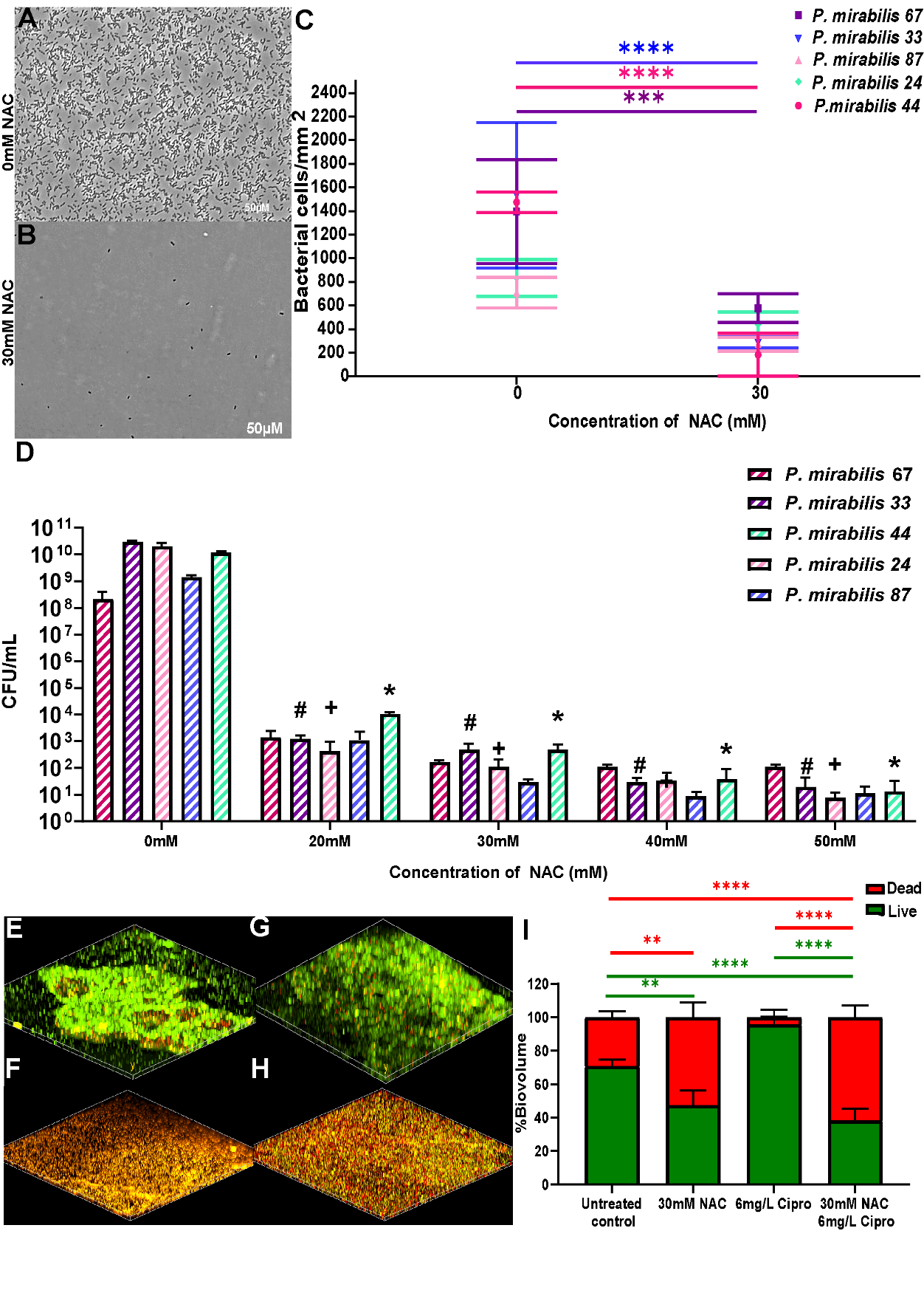


**Supplementary Figure 4 The concentration dependent effect of NAC alone on the preformed biofilms of all tested clinical *P. mirabilis* strains.**

The effect of NAC on bacteria in preformed biofilms was tested at concentrations ranging from 0-50- mM.

**
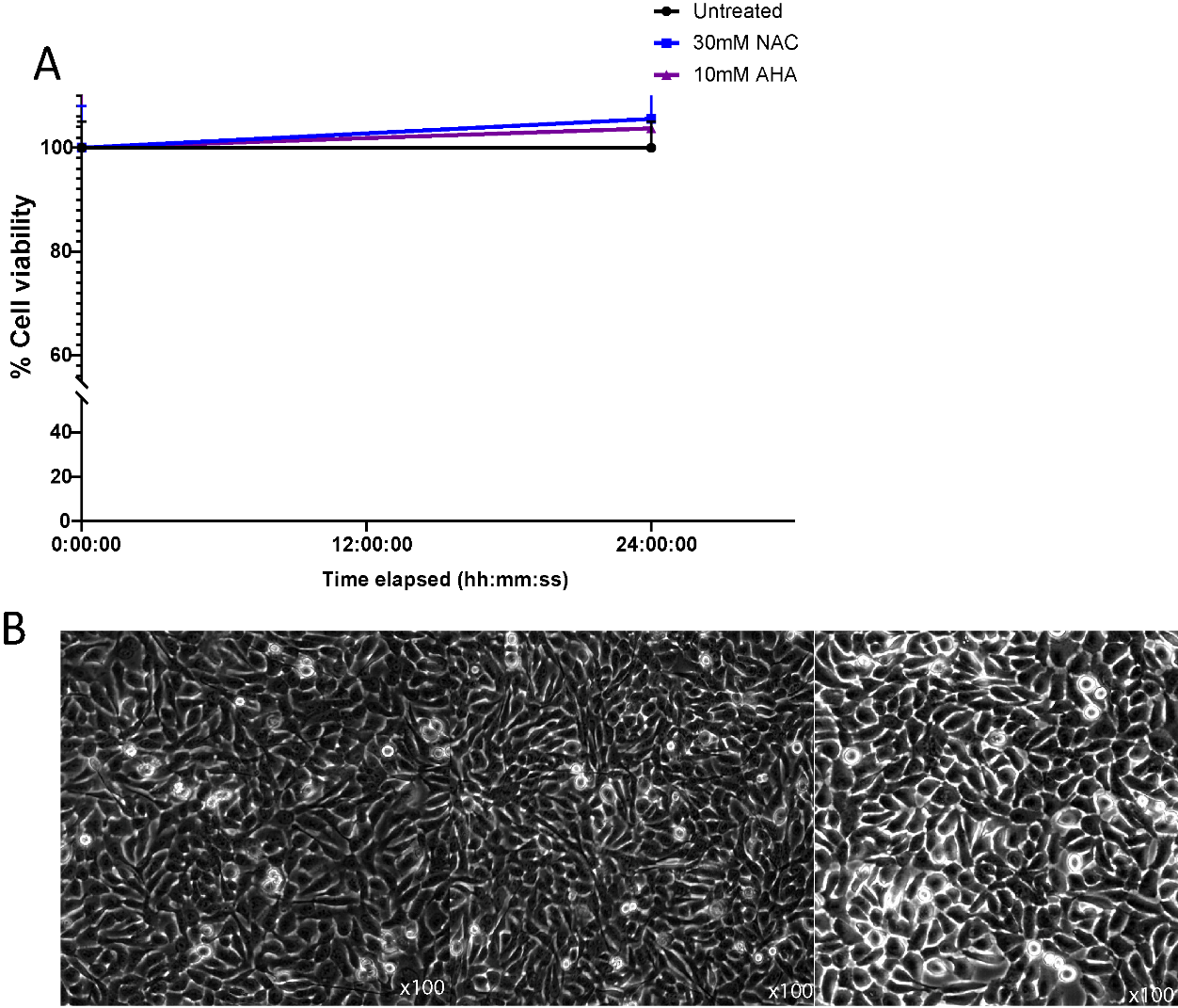
**

**Supplementary Figure 5 The cytotoxicity of NAC on BECs type 5637, tested using ISO method** **British Standard ISO 10993-5:2009 – direct contact assay**

**A** The cytotoxicity of 3 mM NAC was quantified using a direct contact assay method according to the British Standard above. Cytotoxicity for both NAC and AHA was quantified at the 24 h timepoint. **B** Three images of BE cells: untreated, 30mM NAC, 10mM AHA respectively, at the 24 h timepoint.
